# Stabilisierung von Elektronentransferwegen erlaubt Stabilität von Biohybrid‐Photoelektroden über Jahre

**DOI:** 10.1002/ange.202201148

**Published:** 2022-04-19

**Authors:** Vincent M. Friebe, Agata J. Barszcz, Michael R. Jones, Raoul N. Frese

**Affiliations:** ^1^ Fachbereich Physik und Astronomie LaserLaB Amsterdam VU Universität Amsterdam De Boelelaan 1081 Amsterdam 1081 HV Niederlande; ^2^ Fakultät für Biochemie Gebäude für biomedizinische Wissenschaften Universität von Bristol University Walk Bristol BS8 1TD Großbritannien; ^3^ Lehrstuhl für Elektrobiotechnologie Campus Straubing für Biotechnologie und Nachhaltigkeit Technische Universität München Schulgasse 22 94315 Straubing Deutschland

**Keywords:** Biosensoren, Elektrochemie, Photochemie, Photosynthese, Solarenergieumwandlung

## Abstract

Die Nutzung natürlicher photosynthetischer Enzyme in biohybriden Anwendungen stellt eine attraktive und potenziell nachhaltige Möglichkeit zur Umwandlung von solarer Energie in Elektrizität und Brennstoffe dar. Jedoch begrenzt die Stabilität von photosynthetisch aktiven Proteinen nach der Implementierung in biohybride Anwendungsdesigns die operative Lebensdauer von Biophotoelektroden auf bisher wenige Stunden. In dieser Publikation demonstrieren wir, wie sich die Stabilität einer mesoporösen Elektrode, welche mit dem Photoprotein RC‐LH1 aus *Rhodobacter sphaeroides* beschichtet ist, erheblich steigern lässt. Durch die Aufrechterhaltung der Elektronenübertragungswege konnte die operative Lebensdauer unter Dauerlicht auf 33 Tage gesteigert werden und die operative Funktionalität nach einer Lagerung über mehr zwei Jahre hinweg demonstriert werden. Kombiniert mit hohen Photoströmen, die Spitzenwerte von 4.6 mA cm^−2^ erreichten, erzeugte die optimierte Biophotoelektrode eine kumulative Leistung von 86 C cm^−2^, die höchste bisher berichtete Leistung für diese Art von Elektroden. Unsere Ergebnisse zeigen, dass der Faktor, welcher die Stabilität einschränkt, die Architektur der Struktur ist, die das Photoprotein umgibt, sowie das entsprechende biohybride Sensoren und photovoltaische Geräte mit einer Betriebsdauer von mehreren Jahren möglich sind.

## Graphical abstract

Die Anwendung von biohybriden Technologien wird in erster Linie durch die Instabilität der biologischen Komponente eingeschränkt. Hier verlängern wir die Betriebsstabilität von Photoproteinen, die an mesoporöse ITO‐Elektroden adsorbiert sind, auf 33 Tage bei intensiver Belichtung und 28 monate bei Lagerung, was die Umsetzbarkeit von proteinbasierten Geräten über Jahre hinweg demonstriert.

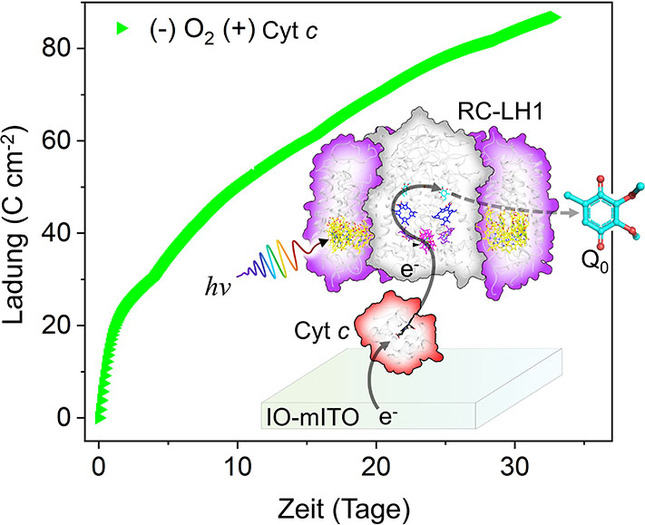

## Einführung

Der Großteil der Biosphäre bezieht ihre Energie durch die Umwandlung von Sonnenenergie, die von nanoskaligen Photodioden, den so genannten Reaktionszentren (RCs), katalysiert wird. Diese Pigmentproteine, die sowohl in Eukaryoten als auch in Prokaryoten vorkommen, verwenden entweder Chlorophyll (Chl) oder Bakteriochlorophyll (BChl), um energiereiches Sonnenlicht einzufangen, umzuwandeln und die Energie letztlich zu speichern.^[1]^ Obwohl die Photosynthese insgesamt kein besonders energieeffizienter Prozess ist,^[2]^ finden die primären Vorgänge der Energieübertragung beim Einfangen des Lichts und der Ladungstrennung bei der Energiestabilisierung mit sehr hohen Quanteneffizienzen statt (Vorgänge pro absorbiertem Photon).^[3]^ Die biomolekularen Grundlagen dieser hohen Effizienz haben das Design von künstlichen Materialien für die nachhaltige Umwandlung von Lichtenergie beeinflusst,[^3^, ^4^] und auch das Interesse an der direkten Integration photosynthetischer Komplexe in biohybride technische Anwendungen gestärkt.[^5^, ^6^, ^7^, ^8^]

Die drei Arten von Photoproteinen, die am häufigsten für die Herstellung von Biohybridelektroden verwendet werden, sind die Chl‐haltigen Komplexe des Photosystems II (PSII) und des Photosystems I (PSI) aus oxygenen Cyanobakterien und Pflanzen sowie die BChl‐haltigen Komplexe des Reaktionszentrums‐Lichtsammelkomplexe 1 (RC‐LH1) aus anoxygen photosynthetischen Bakterien wie *Rhodobacter* (*Rba*.) *sphaeroides*.^[9]^ Jüngste Fortschritte, einschließlich der Entwicklung von transparenten Elektroden mit großer Oberfläche, die eine erhebliche Beladung mit Photoproteinen und eine hohe Lichtdurchdringung ermöglichen,[^10^, ^11^, ^12^, ^13^, ^14^, ^15^] haben zu einigen der leistungsstärksten semi‐künstlichen Biophotoelektroden geführt, die es derzeit gibt. Beispiel hierfür sind PSII, das auf einer inversopalen, mesoporösen Indiumzinnoxid‐Elektrode (IO‐mITO) immobilisiert wurde und Photostromdichten von bis zu 930 μA cm^−2^ erzeugte,[^10^, ^14^] sowie PSI, das mit Cytochrom (Cyt) *c* mit einer IO‐mITO‐Elektrode verdrahtet. wurde und Photoströme von 300 μA cm^−2^ aufwies.^[15]^ Auf nanostrukturiertem Silber aufgebrachte RCs von *Rba. sphaeroides* haben in einer herkömmlichen biophotoelektrochemischen Zelle Photoströme von bis zu 416 μA cm^−2^ erzeugt,^[11]^ während RC‐LH1‐Proteine, an photoaktive Halbleitermaterialien in fester Form gekoppelt, stabile Photoströme von bis zu 1.3 mA cm^−2^ aufwiesen.^[16]^ RC‐LH1‐Multischichten, die zuvor mit Gelelektrolyten getränkttränkt wurden, haben wiederum stabile Photoströme von bis zu 850 μA cm^−2^ gezeigt.^[17]^ Obwohl diese Stromdichten zwar immer noch eine Größenordnung von denen von Solarzellen aus rein synthetischen Materialien entfernt sind, wird dieser Unterschied durch die rasche Verbesserung der Leistung von Biophotoelektroden zunehmend geringer.[^6^, ^14^, ^18^]

Das Haupthindernis für eine breitere Entwicklung von proteinbasierten Anwendungen zur Nutzung von Solarenergie ist die begrenzte Stabilität der Biophotoelektrode im Betrieb und während der Lagerung.[^18^, ^19^] Entsprechende *Reviews* über Systeme auf der Grundlage von PSI^[18]^ und PSII[^14^, ^20^] fassten zusammen, dass die Stabilität im Betrieb, soweit berichtet, nicht über einige Stunden hinausgeht. Der längste beschriebene Dauerbetrieb einer wässrigen Biophotoelektrode auf PSI‐Basis liegt bei 16 Stunden, mit einer Belichtung während 30 % der Zeit.^[21]^ Hingegen liegen die bekannten Betriebsstabilitäten von Bioelektroden auf Basis von PSII in der Größenordnung von Minuten, wobei die geringere Betriebsstabilität in der durch die Hochpotential‐Wasserspaltungsreaktion verursachten Photodestruktion begründet liegt.^[14]^ Das BChl‐haltige RC von *Rba. sphaeroides* führt die Ladungstrennung bei weitaus geringeren oxidierenden und reduzierenden Potenzialen durch als die Chl‐haltigen PSII‐ bzw. PSI‐Komplexe. Entsprechend wurde berichtet, dass RC‐ und RC‐LH1‐basierte Biohybridsysteme bis zu 65 Stunden lang unter kontinuierlicher Belichtung und Luftexposition operieren können.^[22]^ Trotz dieses Vorteils liegt die Betriebsstabilität immer noch drei bis fünf Größenordnungen unter der gewünschten Stabilität von mindestens einem Jahr.[^18^, ^23^]

Im Rahmen dieser Arbeit haben wir versucht, die Konversion von solarer Energie durch *Rba. sphaeroides* RC‐LH1‐Komplexe (Abbildung S1), die auf einer IO‐mITO‐Elektrode gebunden wurden, von Tagen auf Jahre zu verlängern. Wir haben festgestellt, dass die Aktivität der Biophotoelektrode in erster Linie von der Unversehrtheit der Elektronenübertragungswege zwischen dem Photoprotein und der Elektrode selbst abhängt und dass durch die Stabilisierung dieser Schnittstellen der Betrieb unter intensiver Dauerbelichtung auf bis zu einen vollen Monat verlängert werden kann. Wir zeigen zudem eine beispiellose Stabilität während der Lagerung von über zwei Jahren unter kalten, dunklen Bedingungen, eine Eigenschaft, die für enzymbasierte Anwendungen wie Biosensoren unerlässlich ist. Diese Fortschritte bei der Betriebs‐ und Lagerungsstabilität werden dazu beitragen, anwendbare Technologien auf der Grundlage von Photoproteinen zu realisieren, die eine Betriebsstabilität in der Größenordnung von Jahren erfordern.

## Ergebnisse und Diskussion

**Figure 1:**
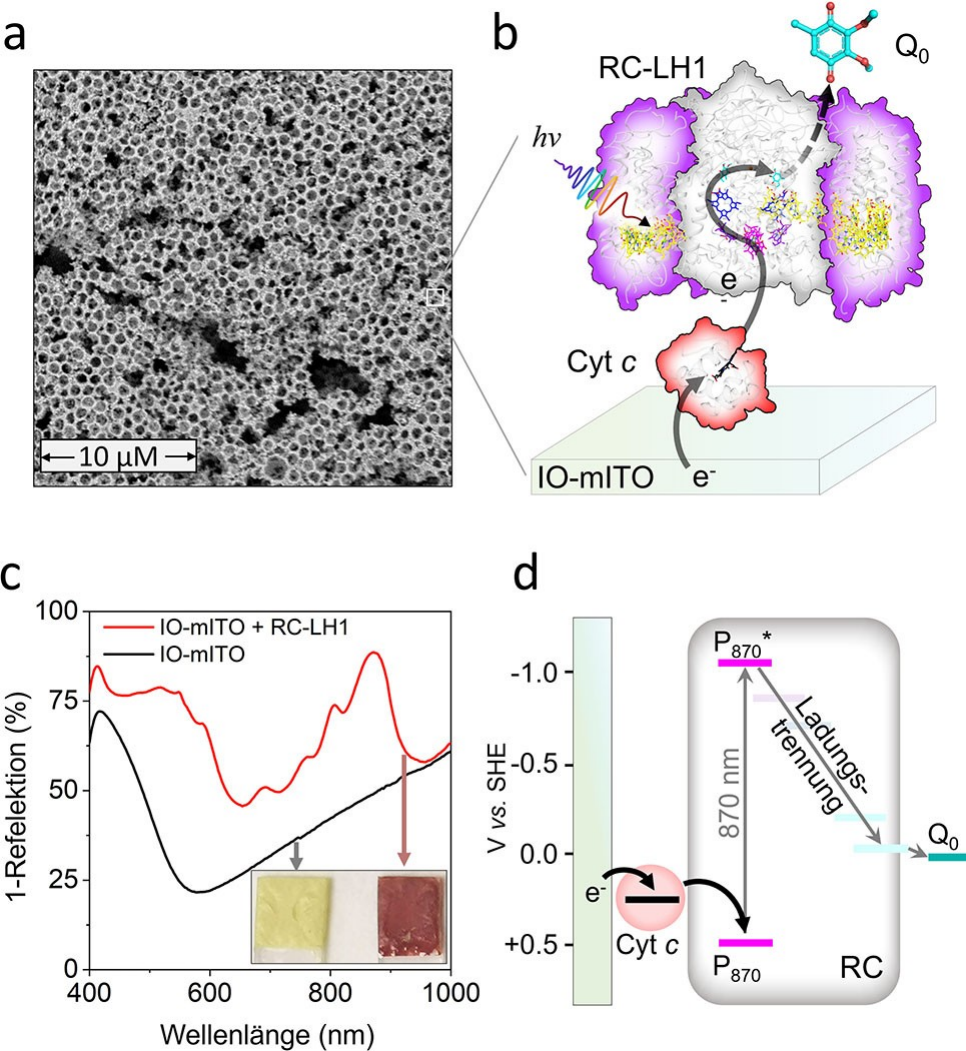
Aufbau, Mechanismus und Präparation der IO‐mITO|cyt *c*|RC‐LH1‐Elektrode. a) REM‐Aufnahme eines Querschnitts durch eine IO‐mITO‐Elektrode aus Polystyrolkügelchen mit einem Durchmesser von 800 nm. Wobei der weiße Balken 10 μm entspricht. b) Schema des Elektronentransferschemas in der RC‐LH1‐Biophotokathode. c) 1‐Reflexionsspektren von IO‐mITO‐Elektroden mit und ohne adsorbierten RC‐LH1‐Protein und (kleines Fenster) Fotos der Elektroden. d) Energieniveaudiagramm des Elektronentransferweges.

Unter Verwendung von ITO‐Glasstreifen als Basis wurden Polystyrolkügelchen mit einem Durchmesser von 800 nm und polydispersive ITO‐Nanopartikeln zur Herstellung von IO‐mITO‐Elektroden verwendet, wie im Abschnitt “Experimente” beschrieben. Eine rasterelektronenmikroskopische (REM) Untersuchung zeigte eine Struktur aus kugelförmigen Hohlräumen mit einem Durchmesser von etwa 800 nm und größeren Spalten (Abbildung 1a). Zur Funktionalisierung mit dem RC‐LH1‐Protein (Abbildung 1b) wurde die Elektrodenoberfläche mit basischer Peroxomonoschwefelsäure‐Lösung behandelt, um eine hydroxylfunktionalisierte,^[24]^ proteinfreundliche Oberfläche^[10]^ zu erzeugen. Anschließend wurde die Elektrode in mehreren Durchgängen zunächst in eine Lösung mit RC‐LH1‐Komplexen und anschließend in eine Lösung mit Cyt *c* getaucht. Die erfolgreiche Adsorption der RC‐LH1‐Komplexe auf der IO‐mITO‐Elektrode konnte mit bloßem Auge verifiziert werden (siehe Abbildung 1c). Die Reflexionsspektren der beschichteten Elektroden zeigten ausgeprägte Signale bei 870, 805 und 600 nm, die den RC‐LH1‐BChls zuzuschreiben sind, und ein breites Signal zwischen 430 und 600 nm, welches den RC‐LH1‐Carotinoiden zugeordnet wird (Abbildung 1c). Die so beschichteten Arbeitselektroden wurden anschließend in einen Puffer mit 1.5 mM Ubichinon‐0 (Q_0_) bei pH 8.0 getaucht. Die Abfolge der Elektronentransferreaktionen, welche durch die Lichtabsorption in diesem System ausgelöst werden, sind in Abbildung 1b zunächst schematisch dargestellt, während Abbildung 1d die Energetik des Vorgangs verdeutlicht. Durch Photoanregung wird der primäre Elektronendonor P_870_ BChl des RC oxidiert und der entsprechende terminale Elektronenakzeptor Q_B_ Ubichinon reduziert (siehe Abbildung S1 für weitere Details). Cyt *c* verbindet die oxidierte Seite des RCs mit der IO‐mITO‐Elektrode, während Q_0_ als mobiler Ladungsträger zur Gegenelektrode dient.[^11^, ^25^, ^26^]

**Figure 2:**
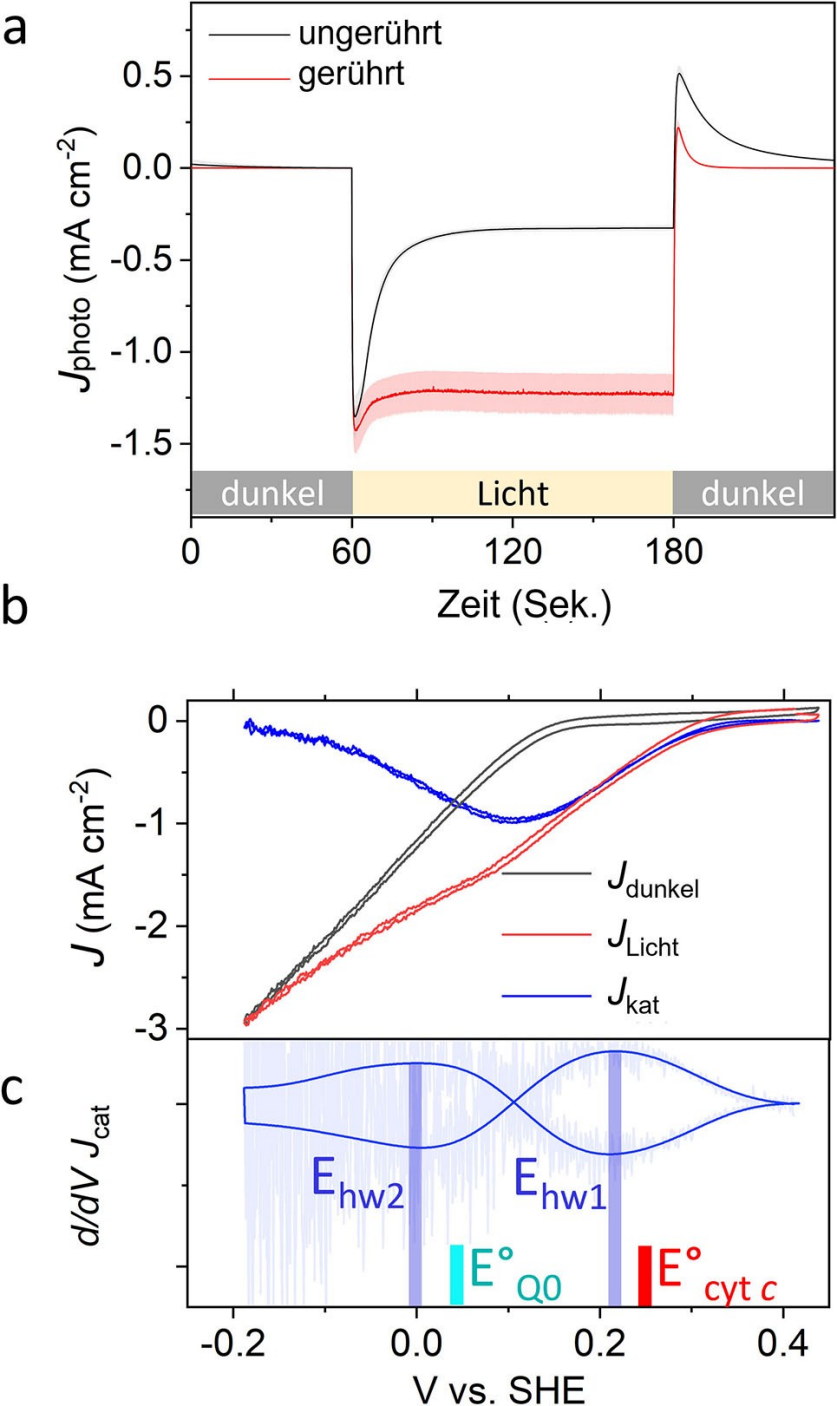
Photochronoamperometrie und photokatalytische Voltammetrie. a) Photostromdichten einer optimierten IO‐mITO|Cyt *c|*RC‐LH1 Elektrode in 20 mM Tris (pH 7.0)/50 mM KCl/5 mM Q_0_ ohne (schwarz) und mit (rot) Rühren. Die schattierten Bereiche zeigen die Standardabweichung, *n*=5. b) Photokatalytische Voltammogramme unter Rühren im Dunkeln (*J*_dunkel_ – schwarz), im Licht (*J*_licht_ – rot) und in der Differenz (*J*_kat (licht‐dunkel)_ – blau). c) Ableitung von *J*_kat_, überlagert mit einer durch Fourier‐Transformation gefilterten Glättung mit einem Cut‐off bei 0.045 Hz, um Schwankungen durch Rühren zu entfernen (dunkelblau). Die Halbwellenpotenziale (*E*_hw_) sind durch die blauen Balken gekennzeichnet. Die Normalpotenziale (*E* ^0^ ) von Q_0_ und Cyt *c* sind durch cyanfarbene bzw. rote Balken gekennzeichnet.

Der durch die Belichtung solcher IO‐mITO|Cyt *c*|RC‐LH1‐Elektroden erzeugte *J*_peak_ wurde als Maß für die Optimierung der Dicke der IO‐mITO‐Schicht und der Menge des geladenen Proteins verwendet. Messungen mit einer bis fünf aufeinanderfolgenden IO‐mITO‐Schichten zeigten, dass drei solcher Schichten und vier bis sechs Runden der Proteinladung optimal waren (Abbildung S2a,b). Diese Konfiguration zeigte einen durchschnittlichen *J*_peak_ von 4.2±0.2 mA cm^−2^, wobei die Elektrode mit der besten Leistung 4.6 mA cm^−2^ erzeugte. Nach diesem anfänglichen Peak fiel der Photostrom stark ab und erreichte ein stabiles Niveau (*J*_stabil_) von circa 0.2 mA cm^−2^ (Abbildung S2c). Da die Umwandlung von solarer Energie eine konstante Leistung unter kontinuierlichen Belichtung erfordert,wurde im Weiteren eine Optimierung von *J*_stabil_ angestrebt. Ein mittlerer Wert für *J*_stabil_ von 0.32±0.03 mA cm^−2^ konnte erreicht werden, indem die Konzentration von Q_0_ auf 5 mM erhöht und der pH‐Wert des Elektrolyt von pH 8.0 auf pH 7.0 gesenkt wurde (Abbildung S3 und Abbildung 2a). Der Anstieg von *J*_stabil_ bei pH 7.0, im Vergleich zu pH 8.0, ist hierbei vermutlich auf das verschobene pH‐abhängige Mittelpunktspotenzial von Ubichinon zurückzuführen (+59 mV pro Abnahme um eine pH‐Einheit).^[27]^ Die Verringerung des pH‐Wertes um eine Einheit senkt effektiv die treibende Kraft für die Ladungsrekombination zwischen Ubichinol und der Elektrode.^[28]^ Diese Pufferbedingungen wurden für die restlichen Untersuchungen dieser Studie beibehalten. Die nachstehenden Daten beziehen sich stets auf eine optimale Elektrode, welche aus drei Schichten IO‐mITO gebildet wurde, was eine ≈69±23 μm dicke Arbeitselektrodenmatrix ergab (Abbildung S2d), und vier Durchgänge der Proteinablagerung.

Ein Beweis dafür, dass der deutliche Abfall von *J*_peak_ zu *J*_stabil_ durch Beschränkungen des Massentransports verursacht wurde, wurde durch Rühren des Puffers erbracht, wodurch dieser Abfall weitgehend aufgehoben wurde (Abbildung 2a und Tabelle 1). Durch das Rühren wurde auch die Rückstromspitze, die bei Beendigung der Belichtung auftrat (Abbildung 2a; bei 180 s), deutlich reduziert, was auf die Ladungsrekombination an der Elektrodenoberfläche zurückgeführt wurde.^[28]^

**Table 1:** Leistung der IO‐mITO|Cyt *c*|RC‐LH1‐Elektrode.

	(−) Rühren	(+) Rühren
*J*_peak_	1367±270 μA cm^−2^	1486±286 μA cm^−2^
*J*_stabil_	322±29 μA cm^−2^	1230±110 μA cm^−2^
Γ_RC‐LH1_	272±27 pmol cm^−2^	272±27 pmol cm^−2^
Γ_Cyt *c* insgesamt_	6.23±0.7 nmol cm^−2^	6.23±0.7 nmol cm^−2^
Γ_Cyt *c* elektroaktiv_	6.15±0.4 nmol cm^−2^	6.15±0.4 nmol cm^−2^
*k*_Cyt *c*_	7.8 s^−1^	
^[a]^TOF (*J*_stabil_)	12.3±2 e^−^ s^−1^ RC^−1^	47±6 e^−^ s^−1^ RC^−1^
^[a]^IQE_app_ (*J*_stabil_)	1.6±0.2 %	6.3±0.8 %

Standardabweichungen beziehen sich auf *n*=4. TOF und IQE_app_ wurden basierenden auf *J*_stabil_ berechnet, wie in Klammern angegeben.^[a]^ Werte sind als scheinbare Werte anzusehen, da Verlustprozesse wie Kurzschluss oder inaktive RC‐LH1s nicht berücksichtigt wurden.

Während *J*_stabil_ unter Rühren hoch war, erreichte die theoretisch RC‐LH1‐Umsatzfrequenz (TOF) einen Wert von nur 47 e^−^ s^−1^ (Tabelle 1), was weit unter der maximal beobachteten TOF von ≈1250 e^−^ s^−1^ in vitro^[29]^ und ≈500 e^−^ s^−1^ in situ^[30]^ liegt (Tabelle S1). Wir nehmen an, dass der relativ niedrige TOF teilweise eine Folge der hohen Proteinbeladung ist, die den einfallenden Photonenfluss ausdünnt, wenn dieser die Elektrode durchdringt, was aufgrund der Selbstbeschattung zu einer niedrigeren durchschnittlichen Anregungsrate der RC‐LH1 führt. Die mittlere RC‐LH1‐Beladung der Elektrode (*Γ*_RC‐LH1_) von 272±25 pmol cm^−2^ wurde durch Lösungsmittelextraktion des BChl a‐Pigments der anhaftenden RC‐LH1‐Komplexe bestimmt (Abbildung S4a). Unter Verwendung des bekannten Extinktionskoeffizienten von RC‐LH1 bei 874 nm,^[30]^ wurde berechnet, dass das geladene Protein 88 % der einfallenden Photonen bei dieser Wellenlänge absorbiert, was mit der gemessenen 1‐Reflexion von 91 % bei derselben Wellenlänge übereinstimmt (Abbildung 1c).

Die Beladung mit RC‐LH1‐Komplexen in der IO‐mITO‐Matrix stellt eine mindestens 20‐fache Steigerung im Vergleich zu den zuvor beschriebenen RC‐LH1‐beschichteten nanostrukturierten Silberelektrode dar,^[11]^ was die 10‐fach höheren Werte für *J*_peak_ im Vergleich zu unserer früheren Arbeit erklärt (Tabelle S1). Die Verteilung von RC‐LH1 auf den Oberflächenschichten der IO‐mITO‐Elektrode wurde mittels konfokaler Fluoreszenzmikroskopie untersucht. Eine Aufnahme eines 10×10 μm großen Bereichs (Abbildung S5a) zeigte Erhebungen und Täler der RC‐LH1‐Fluoreszenz mit einer Größe von etwa 0.8 μm, was mit der Größe der inversen Opal‐Hohlräume übereinstimmt (Abbildung S5b). Es wurde festgestellt, dass die Beladung mit Cyt *c* (*Γ*_Cyt *c*_) im Vergleich zum RC‐LH1‐Komplex einen 20‐fachen molaren Überschuss aufweist (Abbildung S4b). Dieses Verhältnis orientiert sich an früheren Studien, die gezeigt haben, dass ein großer Pool von Cyt *c* erforderlich ist, um Elektronen effizient von einer Elektrodenoberfläche zu den adsorbierten RCs zu leiten.^[25]^

**Figure 3:**
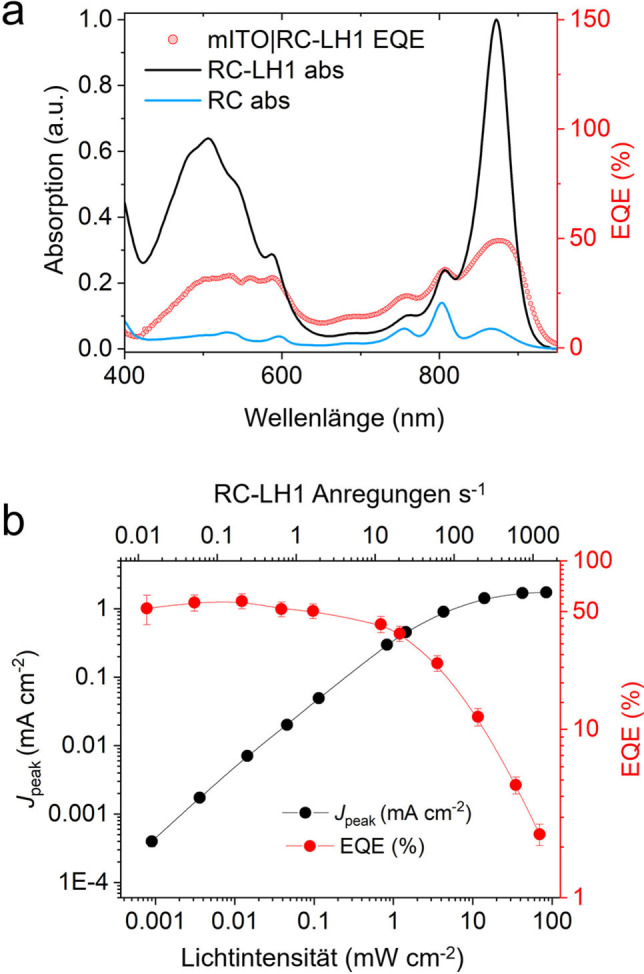
EQE‐Aktionsspektren. a) Prozentuale externe Quanteneffizienz, bestimmt aus Photostromtransienten, die mit 2 nm Inkrementen der Anregungswellenlänge aufgezeichnet wurden, verglichen mit dem RC‐LH1‐Absorptionsspektrum. Das Spektrum eines reinen RC ist zur Unterscheidung der LH1‐Beiträge dargestellt. b) *J*_peak_ und prozentuale EQE als Funktion der Belichtungsintensität und der RC‐Umsetzungsfrequenz (Anregungen s^−1^).

Der in Abbildung 1 dargestellte Elektronentransfermechanismus wurde durch photokatalytische Voltammetrie einer beschichteten Elektrode in einem Elektrolyten unter Rühren verifiziert. Unter Belichtung wurde eine photokathodische Welle (*J*_light_) mit einem Anstieg bei 0.3 V beobachtet, gefolgt von einem zweiten Anstieg bei 0.1 V gegen SHE (Abbildung 2b). Ein Voltammogramm im Dunkeln (*J*_dark_) ergab eine Reduktionswelle, welche von *J*_light_ subtrahiert wurde, um die photokatalytische Welle zu erhalten, welche die lichtgetriebene Elektronentransferreaktion (*J*_kat_) beschreibt. Die Ableitung von *J*_kat_ (Abbildung 2c) ergab zwei Halbwellenpotentiale (*E*_hw_) von +0.22 V und 0.0 V gegen SHE, in Übereinstimmung mit den Mittelwertpotentialen (*E*_m_) von Cyt *c* bzw. Q_0_. Eine weitere Verifizierung dieses Mechanismus ergab sich aus einem Wirkungsspektrum der externen Quanteneffizienz (EQE – Ladungsträger/einfallende Photonen). Die Peaks in diesem Spektrum zeigten eine gute Übereinstimmung mit den Banden im Absorptionsspektrum des RC‐LH1‐Komplexes (Abbildung 3a), wodurch dieser als Quelle des Photostroms identifiziert werden konnte. Ein Vergleich des EQE‐Aktionsspektrums mit einem (1‐T) %‐Absorptionsspektrum ergab jedoch erhebliche Unterschiede, die auf die Art des Lichtmanagements innerhalb der Biophotoelektrode zurückzuführen sind (siehe Abbildung S6 und Begleittext für weitere Einzelheiten).

*J*_peak_ und EQE wurden ebenfalls über einen Bereich von aktinischen Lichtintensitäten von 0.0008 bis 80 mW cm^−2^ bestimmt (Abbildung 3b). Die gemessene maximale EQE von 57±6 % bei niedriger Lichtintensität (3.6 μW cm^−2^/874 nm) ist eine der höchsten bisher berichteten EQEs. Dieser Wert verringerte sich bei Belichtungsstärken über 0.1 mW cm^−2^, was vermutlich auf Limitierungen beim Elektronentransfer und Kurzschlussprozesse zurückzuführen ist, die sich bei hohen RC‐Anregungsraten zusätzlich verstärken (siehe Abbildung S6b und Begleittext für weitere Einzelheiten). Diese Verlustprozesse bilden einen Ansatz für rationale Designstrategien zur Verbesserung der Leistung von Biophotoelektroden.

**Figure 4:**
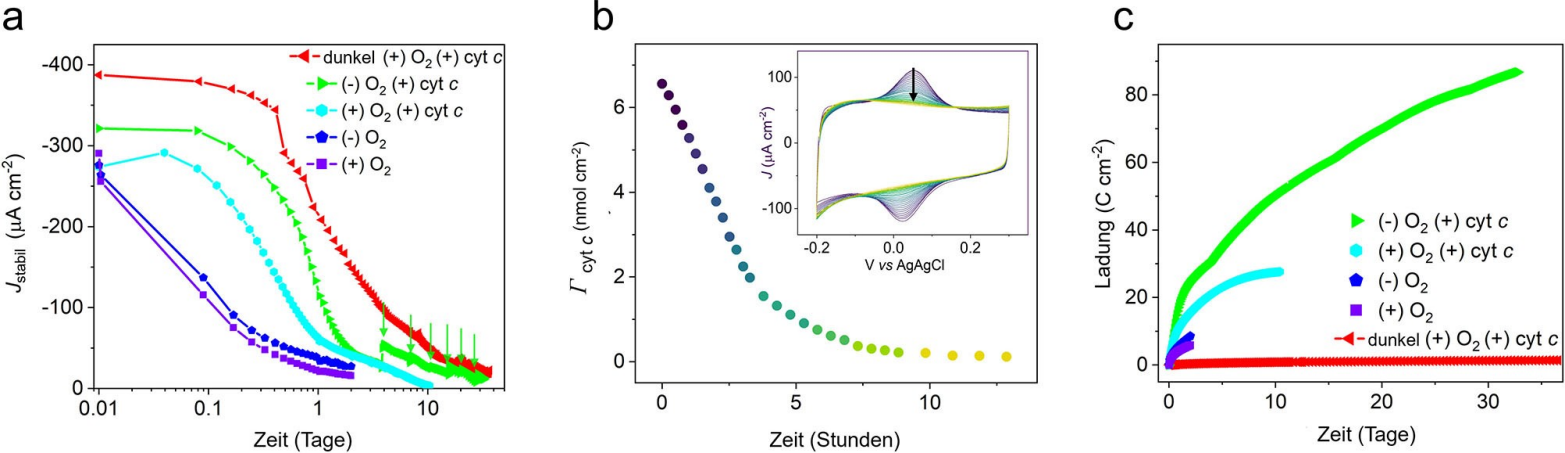
Betriebsstabilität. a) Veränderung von *J*_stabil_ über 33 Tage in Anwesenheit (+) und Abwesenheit (−) von Sauerstoff und Cyt *c*. Bei Dunkelheit wurde ein Photostrom gemessen, wobei das Anregungslicht in den ersten 24 Stunden alle 2 Stunden für 2 Minuten eingeschaltet war (98.3 % Dunkelheit), gefolgt von 2 Minuten alle 4 Stunden (99.2 % Dunkelheit). Ansonsten blieb das Anregungslicht während 95 % der Zeit eingeschaltet. Die Pfeile zeigen den Austausch des Elektrolyten an. Eine lineare Skala ist in Abbildung S11b dargestellt. b) Desorption von Cyt *c*, bestimmt durch Veränderungen der Flächen unter den Peaks des zyklischen Voltammogramms (Inset). Die Scanrate betrug 250 mV s^−1^. c) Kumulativer Ladungstransfer.

Die Betriebsstabilität der IO‐mITO|Cyt *c*|RC‐LH1‐Elektroden wurde unter nahezu kontinuierlicher und intensiver Belichtung (42.3 mW cm^−2^ ) (Zyklen von 57 Minuten Belichtung, 3 Minuten Dunkelheit) in An‐ und Abwesenheit von Sauerstoff untersucht. Im Laufe von 48 Stunden nahm der Wert für *J*_stabil_ sowohl in Anwesenheit (+) als auch in Abwesenheit (−) von Sauerstoff um etwa 90 % ab (Abbildung 4a, violett bzw. blau). Die Abnahmerate war in Gegenwart von Sauerstoff mit einer Halbwertszeit von knapp unter 2 Stunden geringfügig höher als in Abwesenheit von Sauerstoff mit einer Halbwertszeit von über 2 Stunden. Hieraus wurde geschlussfolgert, dass die reversible Desorption des Elektronenübertragungsrelais Cyt *c* von der Elektrode in erster Linie für die Abnahme von *J*_stabil_ unter diesen Bedingungen verantwortlich ist, da die Messungen zeigen, dass die auf der Elektrode geladene Menge Cyt *c* mit einer ähnlichen Halbwertszeit von ≈2 Stunden abnahm (Abbildung 4b). Dies wurde bestätigt, indem der Cyt *c*‐Film am Ende eines solchen zweitägigen Zeitraums durch Eintauchen der Elektrode in eine 200 μM Cyt *c*‐Lösung für 5 Minuten regeneriert wurde, was 81 % der ursprünglichen *J*_stabil_ wiederherstellte (Abbildung S7a). Bei Elektroden, die nicht zuvor unter Luftausschluss operierten, führte die Regeneration des Cyt *c*‐Films jedoch nur zu einer 65 %igen Wiederherstellung von *J*_stabil_ (Abbildung S7b), was auf eine zusätzliche irreversible Zerfallskomponente hinweist, die wir auf Schäden durch reaktive Sauerstoffspezies (ROS) zurückführen.

Zur Stabilisierung des Elektronenübertragungswegs an der Grenzfläche während des Betriebs, wurden dem Arbeitselektrolyt 20 μM Cyt *c* zugesetzt. Dies verlängerte die Betriebshalbwertszeit von ≈2 Stunden auf ≈9 Stunden in Gegenwart von Sauerstoff (Abbildung 4a, cyan). Die Zugabe von Cyt *c* zum Arbeitselektrolyt verstärkte auch die positive Wirkung des Sauerstoffausschlusses. Entsprechend verlängerte sich die Betriebshalbwertszeit auf ≈20 Stunden. Die unter diesen Bedingungen erzeugten Photoströme, fielen nach 10 Tagen auf 5 μA cm^−2^. Die extrahierten Halbwertszeiten der Betriebsstabilität unter verschiedenen Elektrolytbedingungen sind in Tabelle S2 zusammengefasst.

Absorptionsspektren des Cyt *c‐*haltigen Elektrolyten, die entweder vor (Abbildung S8a) oder nach (Abbildung S8b) 48 Stunden nahezu kontinuierlicher Belichtung in Abwesenheit von Sauerstoff aufgenommen wurden, zeigten eine Verschiebung des Verhältnisses von oxidiertem zu reduziertem Q_0_ zugunsten des Letzteren. Dies deutet darauf hin, dass die Abnahme des RC‐Elektronenakzeptors im Volumenelektrolyten für den Abfall von *J*_stabil_ mitverantwortlich sein könnte. In einem Experiment, bei welchem der Elektrolyt alle paar Tage erneuert wurde, gelang es den Fotostrom teilweise wiederherzustellen und diesen 33 Tage lang unter nahezu kontinuierlicher und intensiver Belichtung auf einem Niveau von 10–20 μA cm^−2^ zu halten (Abbildung 4a, grün). Dies stellt einen neuen Rekord für den Dauerbetrieb einer Biohybrid‐Elektrode auf der Basis eines photosynthetischen Enzyms dar und übertrifft die bisherigen Bestwerte einer PSI‐Biohybrid‐Elektrode, die bei einer Belichtung über ≈30 % der Zeit 16 Stunden lang Aktivität zeigte, um das etwa 50‐fache.^[21]^ Bisherige bRC‐Bestwerte von 65 Stunden werden durch unsere Werte um mehr als das 12‐fache übertroffen.^[22]^ Darüber hinaus betrug die während des aufgezeichneten Betriebs der Elektrode übertragene Gesamtladung 86 C cm^−2^ (Abbildung 4c), 45‐mal mehr als ein früherer Vergleichswert unter Verwendung von RC‐LH1‐Komplexen.^[11]^ Die intensiven Lichtbedingungen (46 mW cm^−2^/870 nm LED) führten zu etwa 2.2 Milliarden Anregungen pro RC‐LH1‐Komplex pro Monat. Dies entspräche der Anzahl an Anregungen, die der Komplex in einem ganzen Jahr Sonneneinstrahlung in den Niederlanden erfahren würde.^[31]^ In Anbetracht der Tatsache, dass die Photoströme bei ca. 15 mW cm^−2^ (Abbildung 3b), was weit über der Sonneneinstrahlung in den Niederlanden liegt, ein Plateau erreichen, vermuten wir, dass die Stabilität unter weniger intensiven Lichtbedingungen weiter verbessert werden könnte, da der bRC die eingehenden Anregungen effizienter in die gewünschte Photochemie umwandeln können sollte, bevor sie in schädlichen Nebenreaktionen verloren gehen.

Um den dem Verlust zugrundeliegenden Mechanismus zu untersuchen, der nicht mit der Photochemie zusammenhängt, wurden die Elektroden im Dunkeln unter aeroben Bedingungen in Anwesenheit von Cyt *c* betrieben. Zur Bestimmung der Photostromaktivität wurden die Elektroden alle vier Stunden einer kurzzeitigen Belichtungsphase ausgesetzt. Hierbei zeigte sich eine ähnliche Abnahme von *J*_stabil_ wie bei kontinuierlicher Belichtung unter anaeroben Bedingungen über den gleichen Zeitraum (Abbildung 4b, rot im Vergleich zu grün). Dies deutet auf einen wichtigen Zersetzungsmechanismus hin, der nicht mit Photokatalyse oder ROS zusammenhängt und den wir auf die Bildung einer isolierenden Ablagerungen auf der Elektrode (*electrode fouling*) zurückführen. Eine solche Elektrodenverschmutzung ist ein häufig auftretendes Problem bei elektrochemischen Biosensoren, das aus der irreversiblen und unspezifischen Adsorption von Proteinen an der Elektrodenoberfläche resultiert[^32^, ^33^] und der Isolierung der Elektrodenoberfläche.^[34]^ In unserem Fall würde die irreversible Elektrodenadsorption von Cyt *c* den schnellen Elektronentransfer an der Grenzfläche zur Elektrode hemmen[^32^, ^35^] und die für die Elektronenübertragung zum RC erforderliche Rotationsmobilität einschränken.^[25]^ Die Annahme einer Elektrodenverschmutzung durch Cyt *c* wird durch die verlangsamten Raten des Einsetzens des Photostroms (Abbildung S9) und des Grenzflächenelektronentransfers (Abbildung S10, weitere Einzelheiten siehe Begleittext) nach 10 Tagen Betrieb in einem Cyt ‐haltigen Arbeitselektrolyt unterstützt.

Eine weitere Bestätigung dieser Annahme lieferten die wiederherstellbaren Photoströme (der Photostrom nach Regeneration des Cyt‐Films), wobei die Photoströme nach 10 Tagen Betrieb um etwa 60 % höher waren, wenn der Arbeitselektrolyt kein Cyt *c* enthielt (Abbildung S11). Letztlich führte die Erneuerung des Elektrolyten zu keiner wesentlichen Verbesserung des Photostroms bei Elektroden, die im Dunkeln betrieben wurden (Abbildung S11b, rote Pfeile), im Gegensatz zu den Ergebnissen für Elektroden unter nahezu kontinuierlicher Belichtung (Abbildung 4b und S11b, grüne Pfeile). Was das Photoprotein betrifft, so wurde im EQE‐Aktionsspektrum nach 30 Tagen Betrieb im Licht ein deutlicher Verlust an funktionellem LH1 (95 %) beobachtet (Abbildung S12a). Diesen führen wir teilweise auf die Entkopplung des LH1 vom RC zurück, da die spektrale Signatur des LH1‐Rings im 1‐Reflexionsspektrum der Elektrode nicht proportional abnahm (Abbildung S12b). Der Verlust an funktionellem LH1 wurde auch bei den Elektroden beobachtet, die 30 Tage lang im Dunkeln betrieben wurde, was wir auf die Selbstbeschattung durch nicht verdrahtete RC‐LH1‐Komplexe zurückführen (siehe Abbildung S12b und begleitenden Text für weitere Details). Der Abbau von LH1‐Cofaktoren könnte ebenfalls zum Verlust des LH1‐Phänotyps beigetragen haben. Bei Pigmentextraktionen wurde die Bildung eines Photoabbauprodukts festgestellt, das wir 3‐Acetyl‐Chlorophyll *a* zuschreiben^[36]^ (siehe Abbildung S13 und begleitender Text).

**Figure 5:**
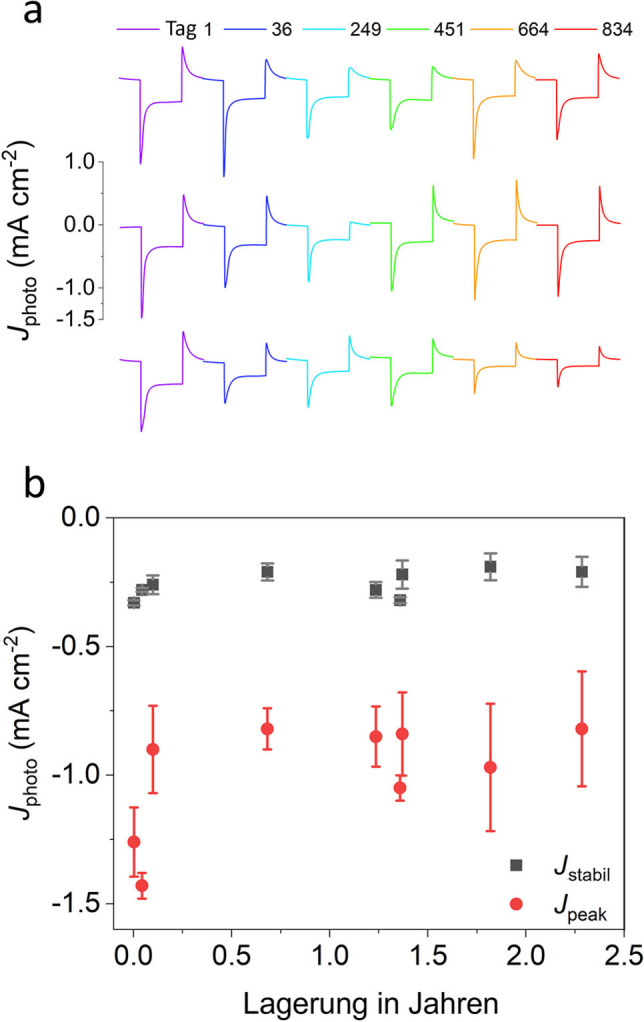
Elektrodenstabilität bei der Lagerung. a) Zwei‐Minuten‐Licht‐an/aus‐Transienten in sechs Intervallen über 834 Tage. Die Elektroden wurden zwischen den Messungen bei 4 °C im Dunkeln in 20 mM Tris (pH 8.0) gelagert. b) Veränderung des Spitzenstroms und des stabilen Photostroms während der Langzeitlagerung über 2.3 Jahre.

Der Photostrom wurde auch in Intervallen für Elektroden gemessen, die im Dunkeln bei 4 °C in einem Cyt *c*‐freien Puffer (20 mM Tris pH 8.0/50 mM KCl) gelagert wurden. Die Ionenstärke des Puffers reichte aus, um den Cyt *c*‐Film während der Lagerung zu desorbieren. Vor der Messung wurde dieser regeneriert. Abbildung 5a zeigt ein Beispiel für Photoströme, die für drei Elektroden in Intervallen (Licht an/aus) über 834 Tagen aufgezeichnet wurden. Obwohl die Spitzenphotoströme der Elektroden in den ersten Monaten der Lagerung um etwa ein Drittel abnahmen, blieben sie danach stabil, und *J*_stabil_ blieb über zwei Jahre der Lagerung bemerkenswert konstant (Abbildung 5b).

Zusammenfassend lässt sich sagen, dass mit frisch präparierten Biophotoelektroden, welche aus einer IO‐mITO‐Matrix bestanden, die mit purpurbaketeriellen RC‐LH1‐Proteinen und Cyt *c* funktionalisiert wurde, relativ hohe Photoströme mit Spitzenphotoströmen von über 4 mA cm^−2^ nach der Optimierung und Dauerphotoströmen von routinemäßig etwa 0.25–0.3 mA cm^−2^ erzeugt werden konnten. Es wurden mehrere Faktoren, welche zu einer Abnahme des Photostroms im Dauerzustand bei Elektroden führen, gefunden und untersucht. Über einen Zeitraum von mehreren Stunden schien die Desorption von Cyt *c* aus der IO‐mITO‐Matrix ein relativ schnelle Abnahme von *J*_stabil_ zu verursachen, die durch die Zugabe von Cyt *c* zum Elektrolyten vermindert werden konnte, wodurch die elektrische Verbindung zwischen den photoaktiven RC‐LH1‐Komplexen und der IO‐mITO‐Elektrode stabilisiert wurde. Der Ausschluss von Sauerstoff war ebenfalls von Vorteil, insbesondere wenn auch Cyt *c* vorhanden war. Ebenso half die Erneuerung des Elektrolyten ein maximales Verhältnis von oxidiertem/reduziertem Chinon für die Ladungsaufnahme aus den RC‐LH1‐Komplexen wiederherzustellen und aufrechtzuerhalten.

Im Laufe eines Monats sank *J*_stabil_ jedoch kontinuierlich auf etwa 5 % des ursprünglichen Wertes. Dieser Rückgang war sowohl bei Elektroden unter nahezu kontinuierlicher Belichtung (95 % der Zeit) als auch bei Elektroden mit nahezu kontinuierlicher Dunkelheit (99.17 % der Zeit) zu beobachten, wenn auch mit leicht unterschiedlichen Anfangsraten (Abbildung 4a). Dies deutet darauf hin, dass ein lichtunabhängiger Prozess während dieser Zeit limitierend wurde. Ein möglicher Kandidat für diesen Prozess ist die allmähliche Inaktivierung des Cyt *c*‐Films. Obwohl gezeigt wurde, dass die Zugabe von Cyt *c* zum Puffer den raschen anfänglichen Abfall von *J*_stabil_ am ersten Tag teilweise abmildern konnte, ist es auffällig, dass der sehr charakteristische Cyt *c*‐Peak, der bei 550 nm in den 1‐Reflexionsspektren frischer Elektroden (Abbildung 1c), und der entsprechende Tiefpunkt, der in den EQE‐Spektren der Photoströme frischer Elektroden (Abbildung 3a) zu sehen war, in den entsprechenden Spektren von Elektroden, die 10 oder 30 Tage lang in Betrieb waren, nicht vorhanden ist (Abbildung S12). Dies könnte auf einen allmählichen und irreversiblen Verlust der Cyt *c*‐Schicht in der Elektrodenmatrix hinweisen, obwohl Cyt *c* in Lösung vorhanden ist. Zusammen mit dem verlangsamten Einsetzen des Photostroms und den verlangsamten Elektronentransferraten an der Grenzfläche sowie der verringerten Cyt *c*‐Beladung nach längerem Betrieb in Gegenwart von wässriger Cyt *c*‐Lösung (Abbildung S10), deuten diese Beobachtungen auf den Verlust von Elektronentransferwegen hin, welcher durch Elektrodenverschmutzung entsteht und als Haupteinschränkung für den Dauerbetrieb aufgeführt werden kann.

Das vielleicht ermutigenste Ergebnis war, dass der Wert für *J*_stabil_ dieser Elektroden über zwei Jahre der Lagerung hinweg im Wesentlichen unverändert blieb (Abbildung 5b), und zwar bei einem Wert von etwa 0.25 mA cm^−2^ . Diese, von uns beschriebene, Lagerungsstabilität übertrifft frühere Bestwerte für die Stabilität von PSI‐Biohybriden ohne jeglichen Aktivitätsverlust bei intermittierenden Tests um mehr als 85 Tage.^[37]^ Auch übertrifft sie die Aufzeichnungen über die biophotovoltaische Lagerstabilität, die über 281 Tage mit Photoströmen um 3 μA cm^−2^ gemessen wurden.^[38]^ Frühere Studien, in denen bRCs mit bloßen Metallelektroden unter Verwendung von Cyt *c* verdrahtet wurden, litten vermutlich unter einem zeitlich beschleunigten Versagen des ET‐Wegs an der Grenzfläche aufgrund der Verschmutzung der Elektrode durch Cyt *c*, das im Laufe der Zeit nachweislich stark an blanke Metallelektroden in nicht‐funktionalen Ausrichtungen adsorbiert^[35]^ oder sogar denaturiert.[^32^, ^33^] Die Verwendung einer hydroxylfunktionalisierten mITO‐Elektrode sowie die Desorption von Cyt *c* von der Elektrodenoberfläche spielten wahrscheinlich eine entscheidende Rolle bei der Verhinderung von Elektrodenverschmutzung während der Lagerung. Diese Stabilitäten deuten darauf hin, dass solche Biohybridelektroden – obwohl sie noch nicht für Anwendungen geeignet sind, die eine längere Lichtexposition erfordern – für Anwendungen geeignet sein könnten, die eine kurzfristige Exposition über einen Zeitraum von Minuten oder ein paar Stunden erfordern. Ein potenzieller Kandidat könnte hierbei die Sensorik sein. Das Potenzial lichtbetriebener RC‐ und RC‐LH1‐Elektroden für Anwendungen in der Herbizid‐Biosensorik[^39^, ^40^, ^41^] und Berührungssensorik[^42^, ^43^] werden derzeit erforscht. Für solche Anwendungen sind zwar nicht unbedingt Elektroden mit langfristiger Photostromstabilität erforderlich, da es sich um relativ kurze Messungen handelt, aber sie erfordern Elektroden, die vor der Verwendung über einen längeren Zeitraum gelagert werden können.[^19^, ^44^] Die in Abbildung 5b zusammengefassten Daten sind in dieser Hinsicht vielversprechend.

Zum Betrieb unter anspruchsvolleren Bedingungen können mehrere Verbesserungen des Elektrodendesigns in Erwägung gezogen werden. Die IO‐mITO‐Matrix diente jahrelang als Träger für funktionelle Photoproteine. Obwohl sie anfänglich sehr effektiv war, erwies sich die Verwendung von Cyt *c* zur “Verkabelung” der RC‐LH1‐Komplexe mit der Elektrode als problematisch. Eine robustere Verdrahtungseinheit, die der Stabilität der Photoproteine entspricht, wäre zweifellos von Vorteil; insbesondere, wenn sie die Elektrode nicht verschmutzen würde. Des Weiteren wäre es von Vorteil, die Stabilität der Photoproteine selbst zu untersuchen. Wie in Abbildung 4a dargestellt, bestünde eine Möglichkeit darin, Sauerstoff auszuschließen, da dessen Anwesenheit den Stromabfall im Laufe der Zeit in unseren Messungen beschleunigte, was mit früheren Ergebnissen übereinstimmt.^[21]^ Eine andere Möglichkeit wäre die Verwendung von “nackten” RCs anstelle der größeren RC‐LH1‐Komplexe. Obwohl letztere eine höhere Lichtsammelkapazität haben und tendenziell höhere Photoströme erzeugen, scheint das LH1‐System anfälliger für Photoschäden zu sein. Daher könnte eine Option darin bestehen, höhere Dichten der “kleineren, aber widerstandsfähigeren” RC‐Photodiode für Anwendungen zu verwenden, die eine längere Exposition der Elektroden gegenüber hohen Lichtintensitäten erfordern, anstatt des größeren, aber anfälligeren RC‐LH1‐Komplexes, der sich für die Umwandlung von Solarenergie in Umgebungen mit relativ geringen Lichtintensitäten entwickelt hat, in denen eine effiziente Lichtsammlung von größter Bedeutung ist. Natürlich kann die Stabilisierung von Elektronentransferwegen, die RC‐LH1‐Anregungen in die gewünschte Photochemie kanalisieren, statt in konkurrierende photochemische Nebenreaktionen, auch den in dieser Arbeit beobachteten RC‐LH1‐Abbau verhindern.

## Zusammenfassung

In dieser Studie untersuchten wir die funktionelle Stabilität eines Photoproteins in einer abiotischen, biohybriden Umgebung. Wir fanden heraus, dass das Photoprotein die Elektronenübertragungswege überlastet, die es mit der Elektrode verbinden. Und dass wir durch die Erhaltung dieser Wege die kontinuierliche Aktivität der Biophotoelektrode auf einen Monat unter intensivem Dauerlicht verlängern können, was ein ganzes Jahr Sonneneinstrahlung in den Niederlanden entspricht. Indem wir die Verschmutzung der Elektroden während der Lagerung verhinderten, konnten wir über 70 % der Elektrodenaktivität über mehr als zwei Jahre aufrechterhalten. Eine solche hochstabile Plattform eignet sich für Biosensoranwendungen, die lange Lagerzeiten, aber kurze Betriebszeiten erfordern. Die Betriebsstabilität in Biophotovoltaikanwendungen, die eine Funktion unter Dauerlicht über Jahre hinweg erfordert, muss jedoch weiter erforscht werden, um den Prozess des “electrode fouling”, die Verschmutzung der Elektrode, in diesen bisher unbekannten Zeitbereichen zu überwinden. Dennoch haben wir gezeigt, dass Photoproteine unter den richtigen Bedingungen über Jahre hinweg in biohybriden Geräten funktionieren können. Diese Erkenntnisse werden dazu beitragen, die rationelle Gestaltung von biohybriden Anwendungen mit den für biotechnologische Anwendungen erforderlichen Leistungen und Stabilitäten zu gestalten.

## Interessenkonflikt

The authors declare no conflict of interest.

## Supporting information

As a service to our authors and readers, this journal provides supporting information supplied by the authors. Such materials are peer reviewed and may be re‐organized for online delivery, but are not copy‐edited or typeset. Technical support issues arising from supporting information (other than missing files) should be addressed to the authors.

Supporting Information

## References

[1] R. Croce , H. van Amerongen , Nat. Chem. Biol. 2014, 10, 492–501. 24937067 10.1038/nchembio.1555

[2] R. E. Blankenship , D. M. Tiede , J. Barber , G. W. Brudvig , G. Fleming , M. Ghirardi , M. R. Gunner , W. Junge , D. M. Kramer , A. Melis , T. a Moore , C. C. Moser , D. G. Nocera , A. J. Nozik , D. R. Ort , W. W. Parson , R. C. Prince , R. T. Sayre , Science 2011, 332, 805–809. 21566184 10.1126/science.1200165

[3] E. Romero , V. I. Novoderezhkin , R. Van Grondelle , Nature 2017, 543, 355–365. 28300093 10.1038/nature22012

[4] I. McConnell , G. Li , G. W. Brudvig , Chem. Biol. 2010, 17, 434–447. 20534342 10.1016/j.chembiol.2010.05.005 PMC2891097

[5] S. K. Ravi , S. C. Tan , Energy Environ. Sci. 2015, 8, 2551–2573.

[6] V. M. Friebe , R. N. Frese , Curr. Opin. Electrochem. 2017, 5, 126–134.

[7] F. Milano , A. Punzi , R. Ragni , M. Trotta , G. M. Farinola , Adv. Funct. Mater. 2019, 29, 1805521.

[8] L. M. Cavinato , E. Fresta , S. Ferrara , R. D. Costa , Adv. Energy Mater. 2021, 11, 2100520.

[9] M. R. Jones in Photosynthetic Protein-Based Photovoltaics (Hrsg.: S. C. Tan ), CRC, Boca Raton, 2018, S. 109–140.

[10] D. Mersch , C. Y. Lee , J. Z. Zhang , K. Brinkert , J. C. Fontecilla-Camps , A. W. Rutherford , E. Reisner , J. Am. Chem. Soc. 2015, 137, 8541–8549. 26046591 10.1021/jacs.5b03737

[11] V. M. Friebe , J. D. Delgado , D. J. K. Swainsbury , J. M. Gruber , A. Chanaewa , R. Van Grondelle , E. Von Hauff , D. Millo , M. R. Jones , R. N. Frese , Adv. Funct. Mater. 2016, 26, 285–292.

[12] K. P. Sokol , D. Mersch , V. Hartmann , J. Z. Zhang , M. M. Nowaczyk , M. Rögner , A. Ruff , W. Schuhmann , N. Plumeré , E. Reisner , Energy Environ. Sci. 2016, 9, 3698–3709.

[13] X. Fang , K. P. Sokol , N. Heidary , T. A. Kandiel , J. Z. Zhang , E. Reisner , Nano Lett. 2019, 19, 1844–1850. 30689393 10.1021/acs.nanolett.8b04935 PMC6421575

[14] J. Z. Zhang , E. Reisner , Nat. Chem. Rev. 2020, 4, 6–21.

[15] D. Ciornii , A. Kölsch , A. Zouni , F. Lisdat , Nanoscale 2019, 11, 15862–15870. 31380869 10.1039/c9nr04344f

[16] S. K. Ravi , Y. Zhang , Y. Wang , D. K. Nandakumar , W. Sun , M. R. Jones , S. C. Tan , Adv. Energy Mater. 2019, 9, 1901449.

[17] S. K. Ravi , D. J. K. Swainsbury , V. K. Singh , Y. K. Ngeow , M. R. Jones , S. C. Tan , Adv. Mater. 2018, 30, 1704073. 10.1002/adma.201704073 29250868

[18] K. D. Wolfe , D. Dervishogullari , J. M. Passantino , C. D. Stachurski , G. K. Jennings , D. E. Cliffel , Curr. Opin. Electrochem. 2020, 19, 27–34.

[19] H. Zhang , A. M. Carey , K. W. Jeon , M. Liu , T. D. Murrell , J. Locsin , S. Lin , H. Yan , N. Woodbury , D. K. Seo , J. Mater. Chem. A 2017, 5, 6038–6041.

[20] X. Fang , S. Kalathil , E. Reisner , Chem. Soc. Rev. 2020, 49, 4926–4952. 32538416 10.1039/c9cs00496c

[21] F. Zhao , A. Ruff , M. Rögner , W. Schuhmann , F. Conzuelo , J. Am. Chem. Soc. 2019, 141, 5102–5106. 30888806 10.1021/jacs.8b13869

[22] G. J. Magis , M. J. den Hollander , W. G. Onderwaater , J. D. Olsen , C. N. Hunter , T. J. Aartsma , R. N. Frese , Biochim. Biophys. Acta Biomembr. 2010, 1798, 637–645. 10.1016/j.bbamem.2009.12.018 20036635

[23] Q. Fu , X. Tang , B. Huang , T. Hu , L. Tan , L. Chen , Y. Chen , Adv. Sci. 2018, 5, 1700387. 10.1002/advs.201700387 PMC5979782 29876199

[24] X. Hou , X. F. Cheng , J. Zhou , J. H. He , Q. F. Xu , H. Li , N. J. Li , D. Y. Chen , J. M. Lu , Chem. Eur. J. 2017, 23, 16393–16400. 28925062 10.1002/chem.201704059

[25] V. M. Friebe , D. Millo , D. J. K. Swainsbury , M. R. Jones , R. N. Frese , ACS Appl. Mater. Interfaces 2017, 9, 23379–23388. 28635267 10.1021/acsami.7b03278 PMC5520101

[26] V. M. Friebe , D. J. K. Swainsbury , P. K. Fyfe , W. van der Heijden , M. R. Jones , R. N. Frese , Biochim. Biophys. Acta Bioenerg. 2016, 1857, 1925–1934. 10.1016/j.bbabio.2016.09.011 27687473

[27] P. Maróti , C. A. Wraight , Eur. Biophys. J. 2008, 37, 1207–1217. 18351330 10.1007/s00249-008-0301-4

[28] N. Plumeré , M. M. Nowaczyk , Advances in Biochemical Engineering/Biotechnology, Springer, Cham, 2016, S. 127–141. 10.1007/10_2016_7 27475649

[29] F. Comayras , C. Jungas , J. Lavergne , J. Biol. Chem. 2005, 280, 11214–11223. 15632163 10.1074/jbc.M412089200

[30] M. Kamran , J. D. Delgado , V. Friebe , T. J. Aartsma , R. N. Frese , Biomacromolecules 2014, 15, 2833–2838. 24964245 10.1021/bm500585s

[31] A. Calcabrini , H. Ziar , O. Isabella , M. Zeman , Nat. Energy 2019, 4, 206–215.

[32] M. Fedurco , Coord. Chem. Rev. 2000, 209, 263–331.

[33] C. Peng , J. Liu , J. Zhou , J. Phys. Chem. C 2015, 119, 20773–20781.

[34] B. L. Hanssen , S. Siraj , D. K. Y. Wong , Rev. Anal. Chem. 2016, 35, 1–28.

[35] S. Lin , X. Jiang , L. Wang , G. Li , L. Guo , J. Phys. Chem. C 2012, 116, 637–642.

[36] L. Limantara , P. Koehler , B. Wilhelm , R. J. Porra , H. Scheer , Photochem. Photobiol. 2006, 82, 770. 16438618 10.1562/2005-09-07-RA-676

[37] I. J. Iwuchukwu , M. Vaughn , N. Myers , H. O'Neill , P. Frymier , B. D. Bruce , Nat. Nanotechnol. 2010, 5, 73–79. 19898496 10.1038/nnano.2009.315

[38] P. N. Ciesielski , F. M. Hijazi , A. M. Scott , C. J. Faulkner , L. Beard , K. Emmett , S. J. Rosenthal , D. Cliffel , G. Kane Jennings , Bioresour. Technol. 2010, 101, 3047–53. 20064713 10.1016/j.biortech.2009.12.045

[39] S. Trammell , L. Wang , J. M. Zullo , R. Shashidhar , N. Lebedev , Biosens. Bioelectron. 2004, 19, 1649–55. 15142599 10.1016/j.bios.2003.12.034

[40] D. J. K. Swainsbury , V. M. Friebe , R. N. Frese , M. R. Jones , Biosens. Bioelectron. 2014, 58, 172–178. 24637165 10.1016/j.bios.2014.02.050 PMC4009402

[41] M. Chatzipetrou , F. Milano , L. Giotta , D. Chirizzi , M. Trotta , M. Massaouti , M. R. Guascito , I. Zergioti , Electrochem. Commun. 2016, 64, 46–50.

[42] S. K. Ravi , N. Paul , L. Suresh , A. T. Salim , T. Wu , Z. Wu , M. R. Jones , S. C. Tan , Mater. Horiz. 2020, 7, 866–876.

[43] S. K. Ravi , T. Wu , V. S. Udayagiri , X. M. Vu , Y. Wang , M. R. Jones , S. C. Tan , Adv. Mater. 2018, 30, 1802290. 10.1002/adma.201802290 30101422

[44] M. Tucci , P. Bombelli , C. J. Howe , S. Vignolini , S. Bocchi , A. Schievano , Microorganisms 2019, 7, 630. 31795453 10.3390/microorganisms7120630 PMC6956157

