# Supplementary material for: Stabilisierung von Elektronentransferwegen erlaubt Stabilität von Biohybrid‐Photoelektroden über Jahre
Source: Angew Chem Weinheim Bergstr Ger. 2022 Apr 19;134(24):e202201148. doi: 10.1002/ange.202201148 (PMC10947033; doi:10.1002/ange.202201148)
Supplement: Supplementary file 1 — Supporting Information [file ANGE-134-0-s001.pdf]

Angewandte Chemie
Eine Zeitschrift der Gesellschaft Deutscher Chemiker

Supporting Information

Stabilisierung von Elektronentransferwegen erlaubt Stabilität von Biohybrid-Photoelektroden über Jahre

V. M. Friebe, A. J. Barszcz, M. R. Jones, R. N. Frese**

Author Contributions

M.R.J. stellte gereinigte RC-LH1-Komplexe zur Verfügung. A.J.B. und V.M.F. bereiteten die IO-mITO RC-LH1-Biophotoelektrode vor und führten experimentelle Untersuchungen durch. V.M.F. und R.N.F. planten und überwachten die Experimente. Alle Autoren diskutierten die Ergebnisse, schrieben und kommentierten das Manuskript.

Experimental Section

Materials. Horse heart cyt *c*, UQ_0 (2,3-dimethoxy-5-methyl-p-benzoquinone), ammonium hydroxide solution (28%-30%), hydrogen peroxide solution (30%), polystyrene bead solution (10% w/v, 800 nm diameter, LB8-2ML), indium tin oxide nanoparticles (<50 nm diameter, polydispersed) and Tris buffer were purchased from Sigma-Aldrich/Merck GmbH. Indium tin oxide (ITO)-coated glass (CEC007S, 260 nm thick ITO, 7 Ohm/sq, 25 x 25 mm) was purchased from Präzisions Glas & Optik GmbH. Milli-Q water (Millipore, Massachusetts) was used in all procedures. n-Dodecyl-β-D-maltoside (DDM) was purchased from Anatrace Products, LL, Ohio. RC-LH1 pigment proteins were purified as described previously[1].

Fabrication of IO-mITO electrodes. IO-mITO electrodes were prepared as previously reported[2] with modifications as follows. Polydispersed ITO nanoparticles were used as these were recently shown to form an IO-mITO structure that supported a higher peak photocurrent density (J_{peak}) than structures formed from homogenous 10, 20, or 40 nm diameter ITO nanoparticles [3]. ITO-glass slides were cut into 5 x 25 mm strips and sonicated for 15 mins in acetone, 15 mins in ethanol, and 5 minutes in water, and then dried for 5 mins on a hotplate at 100°C. ITO nanoparticles (35 mg) were added to 300 µL of 6:1 methanol:water (v/v) and sonicated for 3 hours at room temperature. An aqueous suspension of 800 nm diameter polystyrene beads was diluted to 2.54% (w/v) in water. The beads were washed with methanol by centrifugation of 1 mL of this 2.54% suspension at 15,000 RCF for 5 mins at RT, decanting the supernatant and resuspending the pelleted beads in 1 mL of methanol. After repeating the methanol wash twice, the pelleted beads were stored in a fridge until use. A 300 µL volume of ITO nanoparticle suspension was added to the pellet of polystyrene beads which were resuspended by stirring with a solid needle, followed by sonication in ice water for 5 mins to obtain a homogenous PS-ITO slurry. A 4.2 µL aliquot of this slurry was drop-cast onto an area of approximately 0.25 cm^2 of ITO conductive glass that was defined using a mask made from clear tape. The tape was removed 5 mins after each drop cast to prevent cracking or chipping of the electrode material at the mask/IO-mITO border. Multiple layers of IO-mITO were built up by allotting a four-hour drying period at 4°C between each drop-casting step, and unless otherwise mentioned all electrodes consisted of three layers. After drop-casting was complete the electrodes were heated to 500°C at a rate of 1°C min^{-1} and annealed at this final temperature for 20 mins. Annealed IO-mITO electrodes were cleaned in a mixture of 30% H_2O_2/H_2O/30% NH_4OH (1:5:1 (v/v)) at 70 °C for 15 min, rinsed with water and heated for 1 h at 180 °C to give a contamination-free hydrophilic ITO surface[2]. Fabrication of IO-mITO|cyt *c*|RC-LH1 biophotocathodes. After cooling to room temperature, cleaned IO-mITO electrodes were incubated for 30 mins in an 8.73 µM solution of RC-LH1 protein which contained PufX in 20 mM Tris (pH 8.0)/0.04% (w/v) DDM. Unbound RC-LH1 complexes were removed by dip-rinsing in 20 mM Tris (pH 8.0) for 1 min. Coated electrodes were then immersed in 200 µM cyt *c* in 20 mM Tris (pH 8) for 5 min, rinsing again in 20 mM Tris (pH 8.0) for 5 mins. This process was repeated for a total of four loading rounds per protein. Unless stated otherwise coated electrodes were stored at 4°C in the dark in 20 mM Tris (pH 8.0).

Scanning Electron Microscopy. A Phenom desktop SEM was used to record images of IO-mITO electrodes that had been cut on the glass side and broken into two pieces.

Photo-chronoamperometry. IO-mITO|cyt *c*|RC-LH1 working electrodes were inserted into a three-electrode photoelectrochemical cell fitted with an Ag/AgCl/3M KCl reference electrode, a platinum counter electrode attached to a Metrohm Autolab Potentiostat (PGSTAT128N, Metrohm Autolab BV, Utrecht, The Netherlands). All measurements were conducted in a buffer comprising 5 mM UQ_0, 50 mM KCl, and 20 mM Tris (pH 7.0) unless stated otherwise. The working electrode was illuminated with near-infrared LED (λ_{peak} = 870 nm, FWHM= 40 nm, I = 42.3 mW cm^{-2}, LED870-66-60, Roithner Lasertechnik GmbH) at a bias potential of +160 *vs* SHE, in ambient air at room temperature unless stated otherwise. The wavelength dependency of EQE was determined as previously described[1]. For light titrations, peak photocurrents were measured at increasing irradiances with an 870 nm LED and a rotating neutral density filter. Control photocurrents recorded before and after the experiment established that degradation during the experimental period was negligible. Stirring was not used due to a notable decrease in electrode performance during the experiment.

Photo-catalytic voltammetry. The biophotoelectrode was measured in an electrochemical cell containing 20 mM Tris pH 7.0, 50 mM KCl and 5 mM Q_0 at a scan rate of 5 mV s^{-1} under forced convection. E_{hw} was determined by identifying the inflection point, or peak of the derivative, of the photocatalytic voltammogram as described previously[1].

Reflectance Spectra. Electrode reflectance was characterized with a Lambda 900 spectrophotometer (Perkin Elmer) fitted with an integrating sphere. The sample was placed in the back position at an 8° tilt in a cuvette filled with 20 mM Tris (pH 8.0).

Pigment extraction for determining RC-LH1 loading & absorptivity (1-T)%. BChl *a* was extracted by the immersion of working electrodes in 80:20 acetone/water (v:v) saturated with Na_2CO_3, followed by sonication on ice for 5 mins in the dark. Denatured protein and scattering ITO fragments were pelleted by centrifugation, and the absorbance spectrum of the supernatant was recorded. RC-LH1 loading was estimated using an extinction coefficient at 770 nm for BChl *a* in 80% acetone of 69.3 × 10^{-3} M^{-1} cm^{-1} and by assuming the RC-LH1 complexes contained 32 molecules of BChl *a*[4] (four in the RC and 28 in the LH1 antenna). The (1-T)% absorptivity spectrum was reconstructed based on the determined loading (272 ± 25 pmol cm^{-2}) which has an absorptivity of 88.4% at 874 nm.

Cyt *c* desorption and loading determination. Cyt *c* was desorbed from the IO-mITO|cyt *c*|RC-LH1 working electrodes by immersing the electrode in 220 µL of 1 M KCl/5 mM potassium phosphate buffer (pH 7.0) for 30 mins[5]. After removal of the electrode, 1M ascorbic acid was added to a concentration of 4.5 µM to reduce the cyt *c* and an absorbance spectrum recorded. The cyt *c* concentration was determined using an extinction coefficient of 29.5 × 10^{-3} M^{-1} cm^{-1} at 550 nm for reduced cyt *c*. Removal of cyt *c* was confirmed by photocurrent and cyclic voltammetry (CV). No desorbed RC-LH1 complexes were detected in the cyt *c* absorbance spectrum.

Calculation of external and internal quantum efficiency and RC-LH1 photon absorption rate. EQE was calculated using:

$$EQE = \frac{j_{pho}}{q\ \phi_{inc}} \qquad (1)$$

where j_{photo} is the peak photocurrent density (C s^{-1} cm^{-2}), q is the elementary charge of an electron (1.602 x 10^{-19} C) and φ_{inc} is the incident photon flux (N_p cm^{-2} s^{-1}) on the electrode surface (N_p is the number of photons). The φ_{inc} of the LED used, centered at 868 nm with a FWHM of 36.5 nm, was calculated to be 3.99x10^{17} N_p cm^{-2} s^{-1} (see Friebe et al.[5] Figure S7 for the broadband φ_{inc} nm^{-1}). We did not modify for scattering.

The apparent internal quantum efficiency (IQE = charge carriers/ absorbed photons) was calculated using:

$$IQE_{app} = \frac{J_{photo}}{q\ \phi_{abs}} \qquad (2)$$

where the total number of absorbed photons (φ_{abs}) corresponded to:

$$\phi_{abs} = \sum_{\lambda=4\ \ nm}^{1000\ nm}(1 - T_{\lambda}) * \Phi_{inc\ \lambda} \qquad (3)$$

where (1-T_{λ}) was the percentage absorption of the RC-LH1 layer at wavelength λ, taken from the RC-LH1 loading determined as described above.

Determination of RC-LH1 turnover frequency and photon absorption rate. The apparent RC-LH1 turnover frequency (TOF_{app}) was determined using:

$$TOF_{app} = \frac{J_{photo}}{n\ F\ \Gamma_{\mathrm{RC-LH1}}} \qquad (4)$$

where j_{photo} is the photocurrent flux in A cm^{-2}, Γ_{RC-LH1} is the RC-LH1 loading in mol cm^{-2}, F is the Faraday constant (96485 C mol^{-1}) and n is the number of electrons per cyt *c* turnover (i.e. one). The apparent RC-LH1 turnover rates assume the quantity and functionality of wired RC-LH1s is 100 %.

Stability measurements. Photocurrents were obtained under near-continuous illumination, whereby the electrode was illuminated for 57 minutes and a dark current baseline was then measured for 3 minutes. The label "(+) cyt *c*" indicates cyt *c* was present in the electrolyte at a concentration of 20 µM which has previously been found sufficient to saturate cyt *c* loading[5], and the label "(-) O_2" indicates the cell was purged with argon to remove oxygen. The electrolyte was refreshed approximately every four days unless otherwise specified. For anoxic experiments, purging the electrolyte with argon was carried out beforehand and an overpressure of argon was maintained in the head space of the electrochemical cell to avoid artifacts from forced convection due to bubbling of argon through the electrolyte solution.

Determination of loading of electroactive cyt *c*. The electroactive surface-bound cyt *c* loading ($\Gamma_{cyt\ c}$) was measured from the area under the CV peak using equation (5), as described previously[5],

$$\Gamma_{cyt\ c} = \frac{Area\ under\ CV\ peak}{nFAv} \qquad (5)$$

where F is the Faraday constant (96485 C mol^{-1}), v is the scan rate in V s^{-1}, A is the electrode area in cm^2, and n=1 for cyt *c*.

Table S1. Performance of RC & RC-LH1 biophotoelectrodes

	**Planar gold|cyt *c*| [b)]RC-LH1 LB film**[ref [6]]	**Nanoporous silver |MUA|cyt *c*| [c)]RC** [ref [5]]	**Nanoporous silver |cyt *c*|RC-LH1**[ref [1]]	**IO-mITO|cyt *c*|RC-LH1 (-) stirring (this work)**
J_{peak}	46 μA cm^{-2}	103 ± 24 μA cm^{-2}	166 ± 13 μA cm^{-2}	1367 ± 270 μA cm^{-2}
J_{stable}	27 μA cm^{-2}	60 ± 10 μA cm^{-2}	80 ± 10 μA cm^{-2}	322 ± 29 μA cm^{-2}
$\Gamma_{RC\text{-}LH1}$	0.93 pmol cm^{-2}	53 ± 12 pmol cm^{-2}	12.1 ± 0.9 pmol cm^{-2}	272 ± 27 pmol cm^{-2}
$\Gamma_{cyt\ c\ electroactive}$	-	298 ± 11 pmol cm^{-2}	-	6.15 ± 0.4 nmol cm^{-2}
$k_{cyt\ c}$	-	41.8 ± 9 s^{-1}	-	7.8 s^{-1}
[a)]**TOF**$_{app}$ (**J_{peak}**)	501.5 e$^-$ s^{-1} RC^{-1}	19 ± 5 e$^-$ s^{-1} RC^{-1}	142 ± 12 e$^-$ s^{-1} RC^{-1}	52 ± 11 e$^-$ s^{-1} RC^{-1}
[a)]**TOF**$_{app}$ (**J_{stable}**)	-	-	-	12.3 ± 2 e$^-$ s^{-1} RC^{-1}
[a)]**EQE**$_{app}$ (**J_{peak}**)	0.28 %	0.31 ± 0.004 %	[d)]0.13 ± 0.004 %	4.7 ± 1 %
[a)]**EQE**$_{app}$ (**J_{stable}**)	-	-	-	1.1 ± 0.15 %
[a)]**IQE**$_{app}$ (**J_{peak}**)	32 %	13 ± 2 %	[d)]39 ± 3 %	7 ± 1.5 %
[a)]**IQE**$_{app}$ (**J_{stable}**)	-	-	-	1.64 ± 0.2 %
**E_{hw} onset**	-	+0.26 vs SHE	+0.22 vs SHE	+0.21 vs SHE
**Stability $t_{1/2}$**	-	-	8 hours	20 hours
**Stability $t_{5\%}$**	-	-	40 hours	30 days
**Light Intensity/colour**	23 mW cm^{-2} / 880 nm LED [e)]	46 mW cm^{-2} / 870 nm LED	100 mW cm^{-2} / 1-sun	42 mW cm^{-2} / 870 nm LED

Standard errors are reported, n=4.
TOF, EQE and IQE are calculated from either J_{peak} or J_{stable}, as indicated in parentheses.
[a)]values are apparent since loss processes such as short-circuiting or inactive RC-LH1s are not considered.
[b)]RC-LH1 contained a tetraheme cytochrome (*R. acidophilus*)
[c)]RC without the accessory light-harvesting 1 complex.
[d)]1-sun illumination (100 mW cm^{-2}) accounts for relatively low EQE in this study due to lower absorption across the entire solar spectrum relative to all other studies that employ an 870 nm LED. This also affects the IQE of this study, which is overestimated due to the low excitation rate.
[e)]LEDs have a FWHM of approximately 50 nm, and their spectral profile is reported in reference [5].

Table S2. Operational stability

Electrolyte	half-life (hrs)
(+) O_2	1.8
(-) O_2	2.1
(+) O_2 (+) cyt *c*	9.4
(-) O_2 (+) cyt *c*	19.7
dark (+) O_2 (+) cyt c	30.5

Half-life corresponds to time where photocurrent has decreased 50%

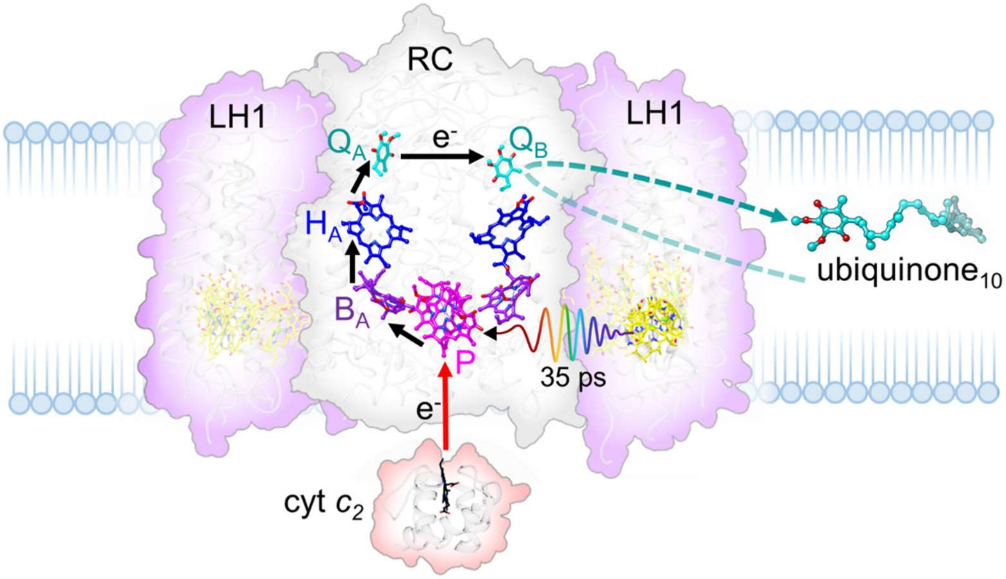

Figure S1. Structure, mechanism and electron transfer interactions of the *Rba. sphaeroides* RC-LH1 complex. The central RC domain is almost fully encircled by the Light Harvesting-1 cylindrical domain to form the so-called RC-LH1 core complex, which is embedded in a phospholipid bilayer membrane[7]. The LH1 domain binds 28 BChl *a* pigments (yellow) and 14 carotenoids (not shown)**,** which significantly enhance the absorption cross-section of the RC. The RC protein coordinates a set of BChl (pink, purple), bacteriopheophytin (BPhe, blue) and ubiquinone-10 (Q_{10}, cyan) cofactors that catalyze photochemical charge separation (black arrows). A pair of BChls forms the primary electron donor (P, magenta) at one side of the membrane, and upon receipt of excited electronic state energy from the LH1 antenna initiates a four-step charge separation that results in the reduction and protonation of hydrophobic ubiquinone-10 at the so-called Q_B site at the opposite side of the membrane[8]. Cofactors B_A, H_A and Q_A are intermediary BChl, BPhe and Q_{10} electron carriers. After double reduction/protonation, the Q_{10} undocks from the Q_B site and diffuses through the membrane interior to the cytochrome *bc*$_1$ complex, where it is oxidized and proton release creates a transmembrane proton gradient, in turn driving ATP synthesis at a solar energy conversion efficiency up to 12%[9]. A water-soluble cyt *c*$_2$ completes an electron transfer circuit by delivering high potential electrons from the cytochrome *bc*$_1$ complex back to the photo-oxidized primary electron donor. Mimicking this natural process, in the present study RC-LH1 complexes have been wired to the IO-mITO electrode using horse heart cyt *c* and to the counter electrode using Q_0, to form biophotoelectrodes capable of cycling light-induced photocurrents when immersed in buffered water solution[5,10–13]. Commercially available horse heart cyt *c* is highly similar in structure and reduction potential to *Rba. sphaeroides* cyt *c*$_2$ and is known to be an effective substitute. Q_0 is a short side-chain hydrophilic analogue of the insoluble native ubiquinone-10, and again is known to act as a highly effective electron transfer mediator in settings employing detergent-purified RC-LH1 complexes in solution[14].

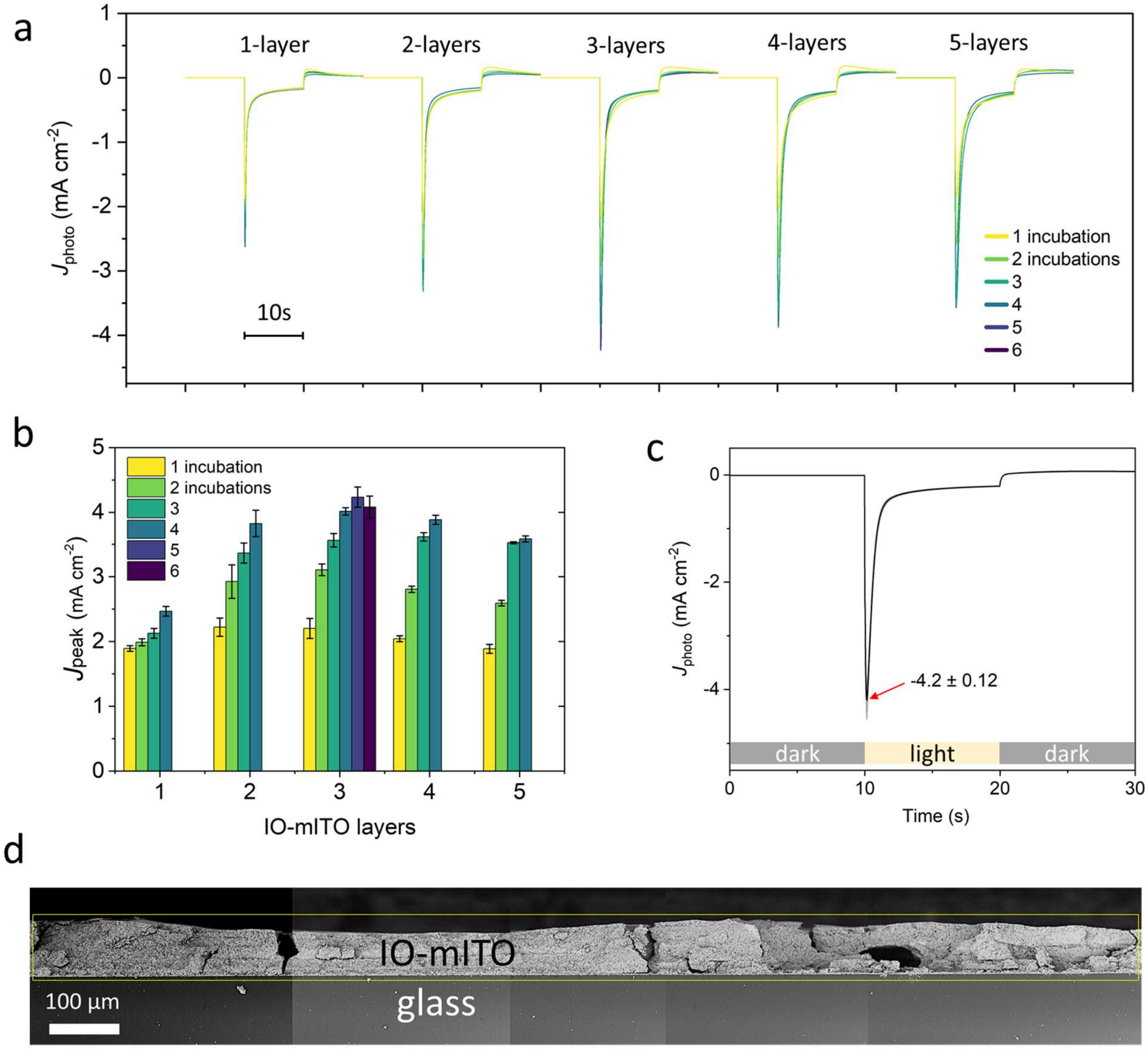

Figure S2. Optimization of IO-mITO thickness and loading. (a) Averaged photocurrent traces as a function of the number of protein loadings for electrodes formed of between one and five successive layers of mITO on FTO coated glass (Sigma Aldrich/Merck 735167). One loading step constituted sequential incubation in RC-LH1 solution for 30 mins, and cyt *c* for 5 mins, with rinsing steps in between. Photocurrents were recorded at a bias potential of +160 mV *vs* SHE. Illumination was performed with an LED centered at 870 nm and an intensity of 42.3 mW cm^{-2}. (b) extracted average peak photocurrents (J_{peak}) from panel a (n=4). (c) Average photocurrent density produced by the best performing 3-layer IO-mITO|cyt c|RC-LH1 working electrode after five protein loading rounds. (d) Merged SEM image of the cross-section of a typical three-layer IO-mITO electrode. The average electrode thickness was 69 ± 23 µm (n = 75).

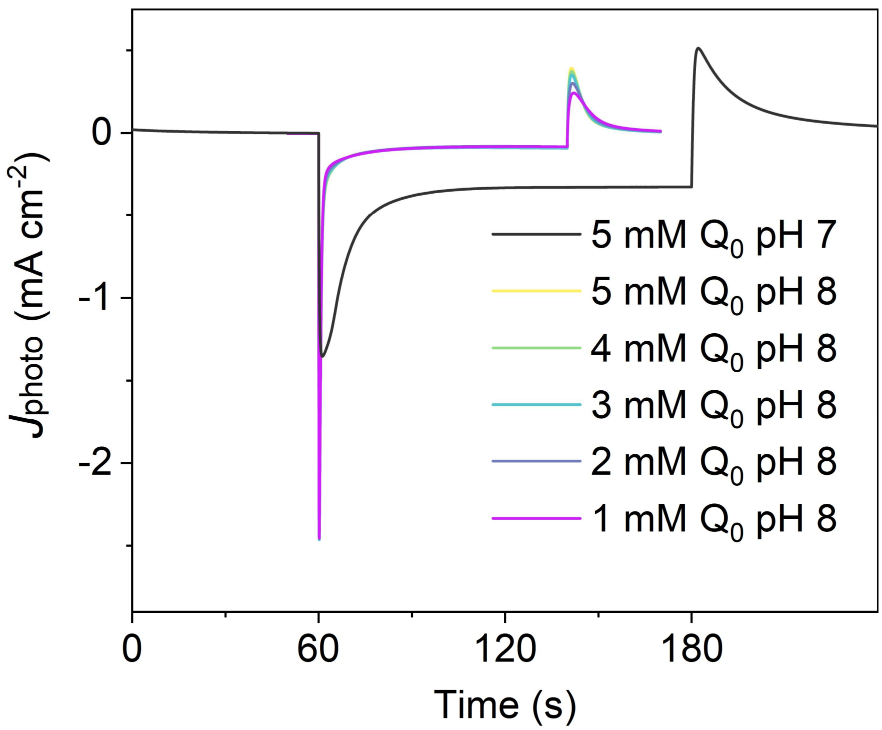

Figure S3. Optimization of quinone concentration and pH for improved J_{stable}. Light is turn on at 60 seconds and turned off at 140 seconds for samples at pH 8, while turned off at 180 seconds for the sample at pH 7.

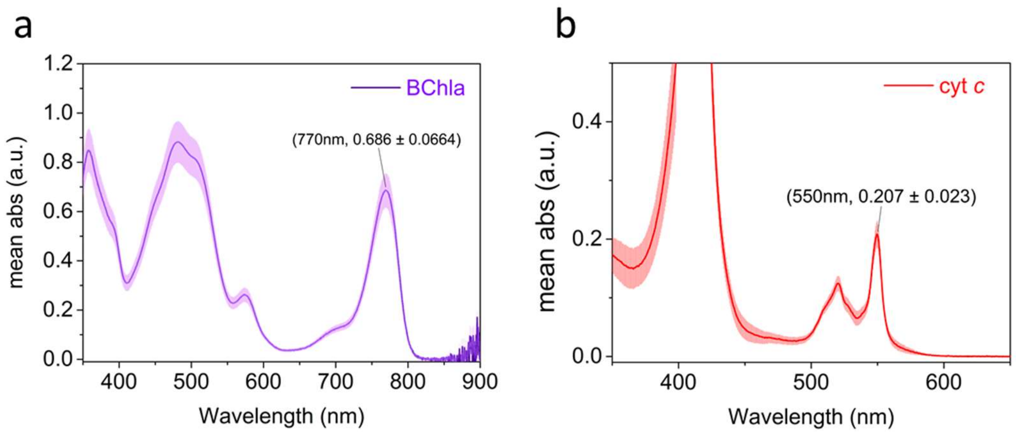

Figure S4. Protein loading. **(a)** Absorbance spectra of BChl *a* extractions from electrodes. **(b)** Absorbance spectra of cyt *c* desorbed from electrodes using 1 M KCl. Error bars are indicated in both plots by the shaded regions, n=3.

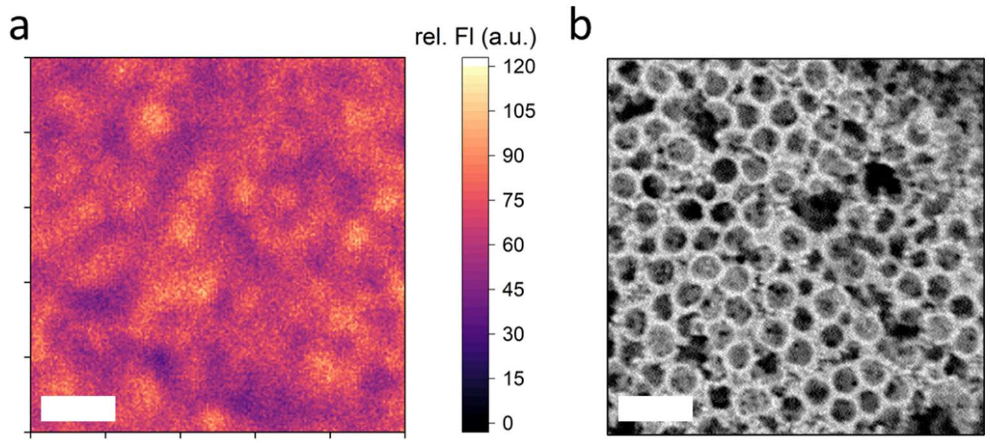

Figure S5. Protein distribution on the electrode. (a) Confocal fluorescence images of IO-mITO|RC-LH1 surfaces. (b) SEM image of an IO-mITO surface. Scale bar represents 2 μm.

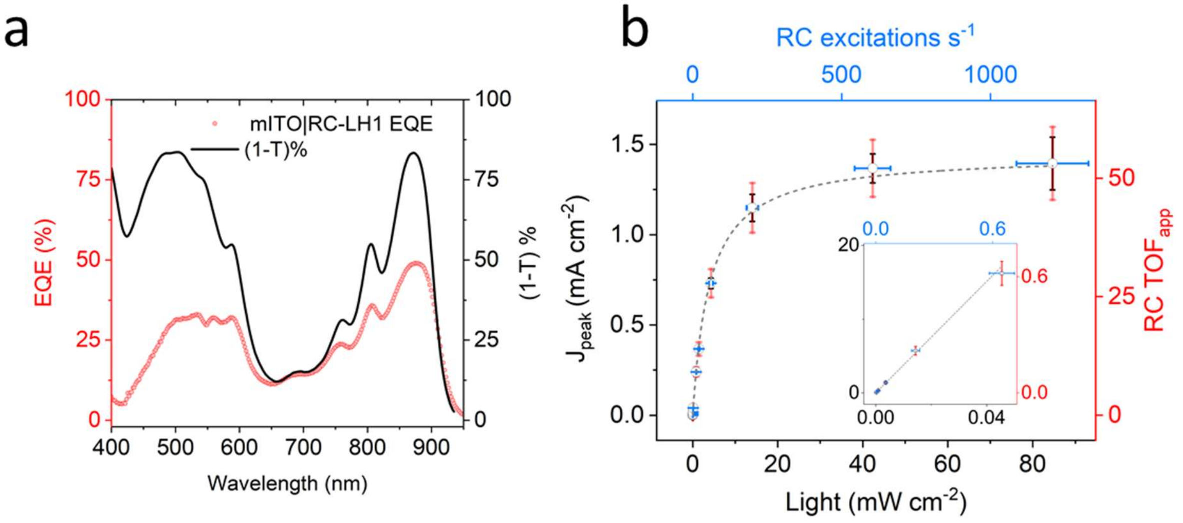

Figure S6. Light harvesting efficiency. (a) Percentage external quantum efficiency compared with an absorptivity (1-T) % calculated from RC-LH1 loading. (b) J_{peak} as a function of illumination intensity. Error bars represent standard deviation (n=3) and are color-coded with their respective axis. The grey dotted line represents a Michaelis-Menten fit of the RC excitations and RC TOF_{app} axis with a V_{max} = 55 ± 2 TOF s^{-1}, K_M= 56 ± 2 excitations s^{-1}, and an R-square of 0.99796. The inset is a zoom of the linear regime, fitted with a slope of 1.01 and Pearson's R of 0.9986

Supporting Text to Figure S6. Comparison between the expected wavelength dependent lighted harvesting capacity, as described by a (1-T) % absorptivity spectrum, and resultant wavelength dependent energy conversion, as described by the EQE action spectrum, revealed significant disparities (Figure S6a). The relatively low EQE below 600 nm is partially attributable to the low quantum efficiency of the RC-LH1 carotenoids coupling to charge separation. Dips in the EQE spectrum at 550 nm and 520 nm stem from the parasitic absorption by cyt *c* and further decreases below 450 nm are attributed to parasitic absorption by quinones and the cyt *c* Soret band. The IO-mITO electrode shows high reflectance from 500 to 800 nm and lower reflectance outside of this window (Figure 1c). This also manifests itself in the EQE, whereby regions of high electrode reflectance boost EQE, but windows of high electrode absorption lower EQE (Figure 3b). The overlapping EQE and (1-T) % from 650-700 nm may indicate a window where the loss of light due to parasitic absorption is offset by the increased RC-LH1 absorption cross-section due to scattering.

In order to explore the photoconversion efficiency of the electrodes, photocurrents were measured as a function of excitation intensity (Figure S6b). Rather than stirring the electrolyte to remove artefacts due to charge recombination with the electrode, J_{peak} was used as the criteria for photoconversion efficiency since its value is nearly identical to J_{stable} in the presence of stirring (Figure 2a). To give insight into the kinetics of RC turnover, the light intensity was converted into RC excitation rate (top axis) and J_{peak} to RC TOF (right axis), both based on the RC-LH1 loading determined by pigment extraction (Figure S4). The curve was fitted with the Michaelis-Menten model to give a maximum RC TOF_{app} of 55 ± 2 $e^- RC^{-1} s^{-1}$ and half-maximum (k_M) of 56 RC-LH1 excitations per second. This finding highlights a limitation in RC turnover and defines an optimal

light intensity that averts futile cycling at a relatively low illumination intensity of 0.1 mW cm^{-2} (Figure 3c). However, the high EQE decreased at higher light intensities due to diffusion limitations (Figure 3c).

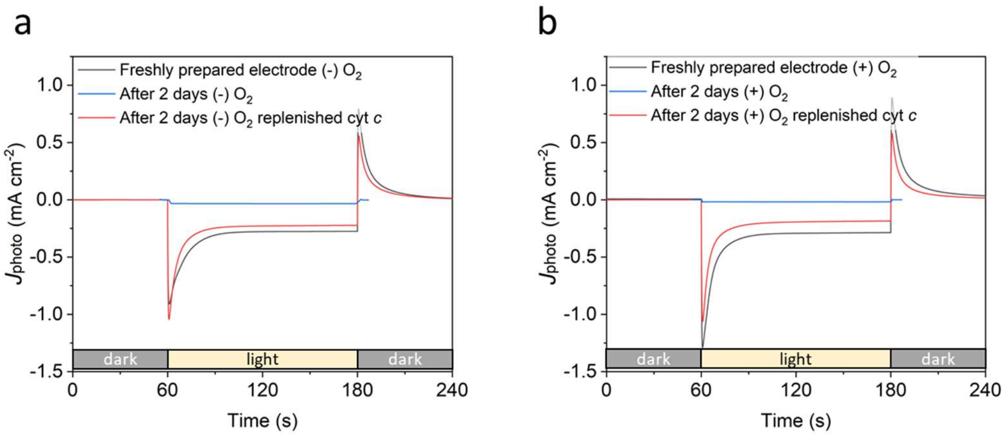

Figure S7. Photocurrent recovery on refreshing cyt *c*. (a) Electrode fatigued in the absence of cyt *c* and absence of oxygen. (b) Electrode fatigued in the absence of cyt *c* and presence of oxygen.

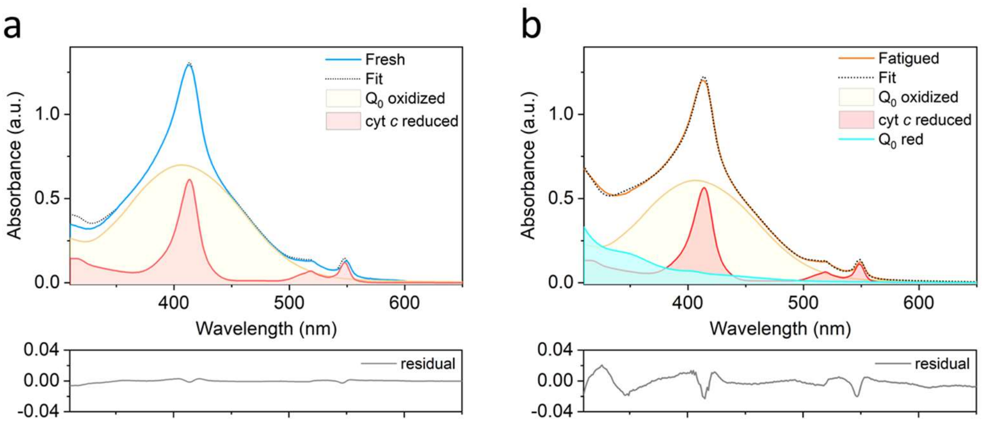

Figure S8. Electrolyte fatigue. (a) Absorbance spectrum of freshly prepared working electrolyte (blue) and deconvolution into components of reduced cyt *c* (red) and oxidized Q_0 (gold). The resulting fit is indicated in dotted black, and the residual of the fitting in the lower panel in grey. (b) Absorbance spectrum of electrolyte fatigued for 2 days in the light (orange) and deconvolution into components of reduced cyt *c* (red), oxidized Q_0 (gold) and reduced Q_0 (cyan). The resulting fit is indicated in dotted black, and the residual of the fitting in the lower panel in grey.

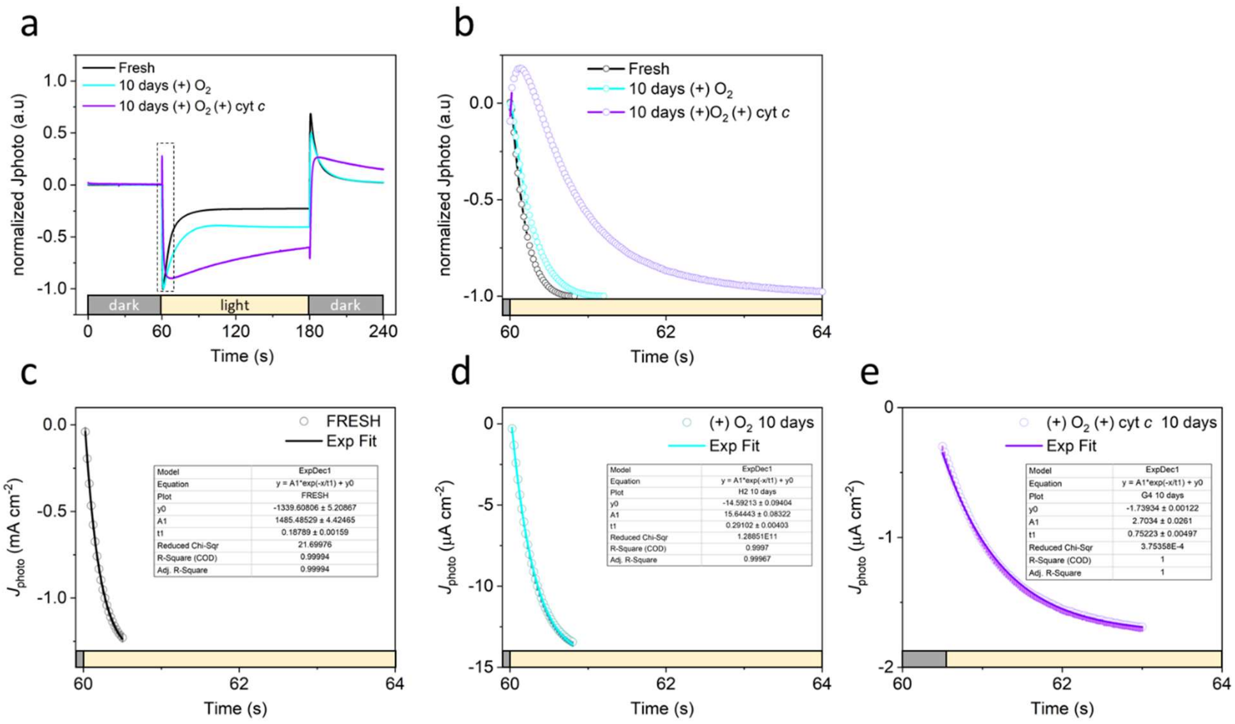

Figure S9. Rate of photocurrent onset. (a) Photocurrents for fresh electrodes and electrodes fatigued for 10 days in air in the absence or presence of cyt *c.* (b) Expanded view of the data in the dotted box in panel a. (c-e) Onset photocurrents were fit to a single exponential decay function, y = y0 + A1*exp(−x/t1), where y is the measured photocurrent at time x, y0 the peak photocurrent and t_1 is the time for the photocurrent to reach 50% of its peak value, as described previously[15]. A single component exponential fit revealed lifetimes of 0.19 s for fresh electrodes, but 0.29 s after 10 days of operation in the absence of cyt *c* and 0.75 s in its presence.

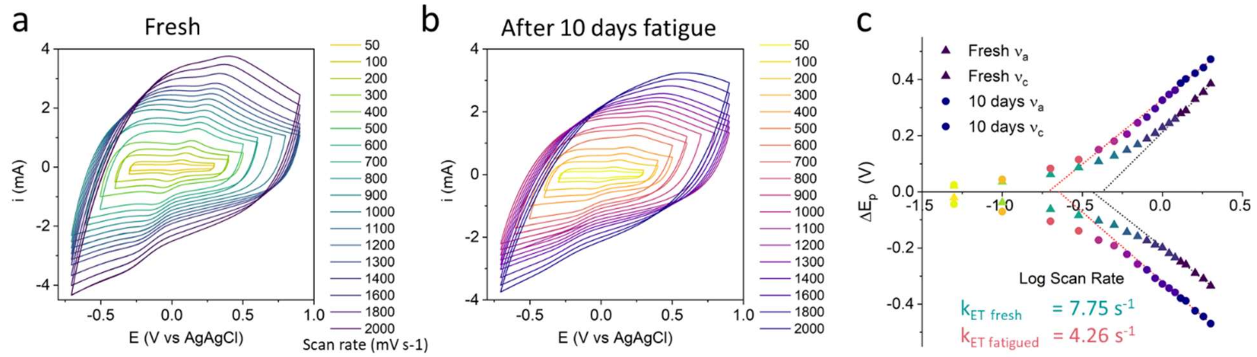

Figure S10. Electrochemical characterization of interfacial electron transfer between IO-mITO and cyt *c*. (a,b) Cyclic voltammograms of electrodes before (a) and after (b) 10 days of incubation in cyt *c*, recorded at scan rates between 50 and 2000 mV s^{-1}. (c) Laviron plots extracted from panels (a) and (b) as previously described[5], depicting peak separation (ΔE_p) vs the log of the scan rate. Linear fits of the last 6 data points with regression to the x-axis intercept value are shown for the fresh electrode (black dotted lines) and fatigued electrode (red dotted lines), representing heterogeneous electron transfer rate constants of 7.75 s^{-1} and 4.26 s^{-1}, respectively.

Supporting text to Figure S10. To assess this kinetic bottleneck further, the rate of electrode-cyt *c* electron transfer was extracted using the Laviron method[16], revealing a decrease in the electron transfer rate constant from 7.75 s^{-1} for a fresh electrode to 4.26 s^{-1} for an electrode after 10 days of operation (Figure S10c). This was accompanied by a reduction in height of cyt *c* peak in cyclic voltammograms and an increase in its width (Figure S10a,b), consistent with less effective interfacial electron transfer due to a loss in integrity of the wiring cyt *c* layer.

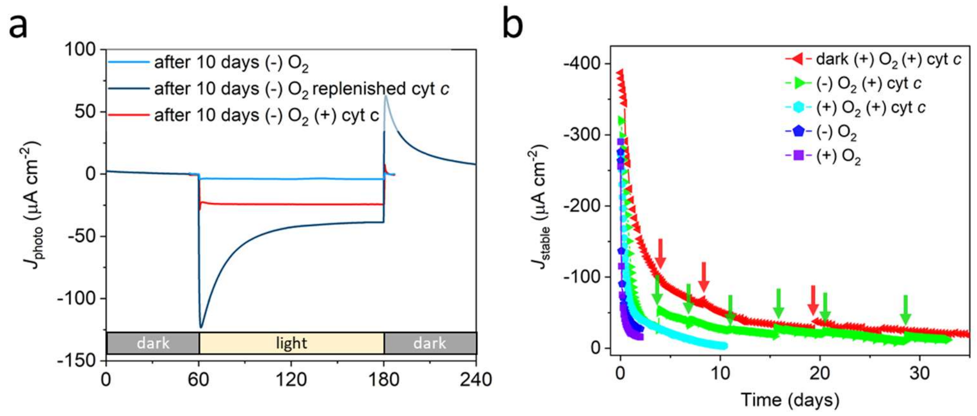

Figure S11. Effect of replenishment of electrode adsorbed cyt *c*. (a) Residual photocurrents for electrodes fatigued for 10 days in anoxic conditions in the absence of cyt *c* (cyan) were much lower than for electrodes fatigued in the presence of cyt *c* (red). Electrodes operated for 10 days in the absence of cyt *c* (cyan) were then immersed in a solution containing 200 μM cyt *c* for 5 mins, rinsed, and then a photocurrent was measured ("replenished cyt *c*" – dark blue). The traces in red and light blue are taken directly from long-term stability experiments (2-hour light on traces) and their X-axis is compressed 57-fold for comparison to the trace in dark blue. Photocurrent onset and off kinetics are thus not accurate for traces in red and light blue. (b) Data on J_{stable} over 33-35 days shown in Figure 4b, plotted on a linear scale. Arrows indicate the replacement of the electrolyte.

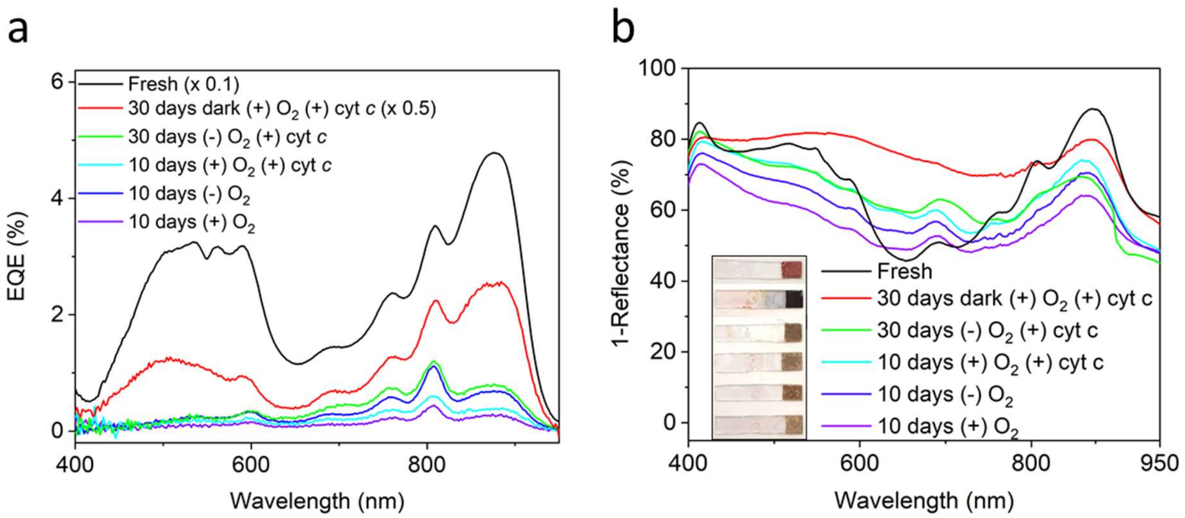

Figure S12. Stability of RC-LH1 complexes and contribution to EQE. (a) Percentage EQE action spectra recorded after 10 or 30 days under various conditions. (b) 1-Reflectance spectra recorded after 10 or 30 days under various conditions, and (inset) images of the electrodes.

Supporting text to Figure S12. EQE action spectra report on changes in the characteristics of the proteins responsible for generating the photocurrent. Action spectra recorded after 30 days of primarily dark conditions showed a ~10-fold decrease in intensity relative to a spectrum recorded for a fresh electrode (Figure S12a), consistent with a 20-fold drop in absolute J_{stable}. However, the line-shape of the spectrum was well-preserved with prominent bands at 760 nm and 805 nm attributable to the RC, a larger band at ~875 nm attributable to the LH1 BChls, and bands at between 400 and 600 nm attributable to the LH1 carotenoids (see absorbance spectra in Figure 3a). In contrast, in EQE action spectra recorded after 10 days of operation under (+)O_2, (-)O_2 or (+)O_2/(+)cyt *c* conditions, and for a 30-day operation under the less destabilizing (-)O_2/(+)cyt *c* conditions, with mostly illuminated conditions for all, there was a disproportionate loss of bands at 875 nm and 420-600 nm attributable to LH1 BChls and carotenoids, respectively, leaving a line-shape similar to the absorbance spectrum of the RC alone (shown in Figure 3a). This indicated that an effect of near-continuous illumination was to decouple the LH1 antenna from the RCs responsible for the residual photocurrent seen after 10 or 30 days. In contrast, the residual photocurrent seen after 30 days of primarily dark incubation was produced by RCs still coupled to their LH1 antenna.

Given that energy transfer is a short-range process, a light-induced decoupling could either stem from a physical detachment of otherwise intact LH1 pigment-proteins from their partner RCs, or degradation of the LH1 pigment proteins (or both). Interestingly, 1-reflectance spectra of electrodes after 10 or 30 days of high-light operation showed only a ~60% decrease in the LH1 absorption band at 875 nm under all conditions (Figure S12b), in contrast to the ~95% decrease in the EQE intensity at this wavelength (Figure S12a). This suggested that the changes in EQE line-shape could not be purely attributed to photodegradation of the LH1 component of the RC-LH1 complex, but might also have involved a loss of energy transfer efficiency between LH1 pigments and the RC. In general, electrodes operated in the presence of oxygen showed greater changes to their EQE spectrum than electrodes operated in the absence of oxygen, despite

similar degrees of retention of the LH1 band in the reflectance spectrum. This suggested larger effects on RC stability and LH1-to-RC connectivity in the presence of oxygen, presumably due to the generation of ROS.

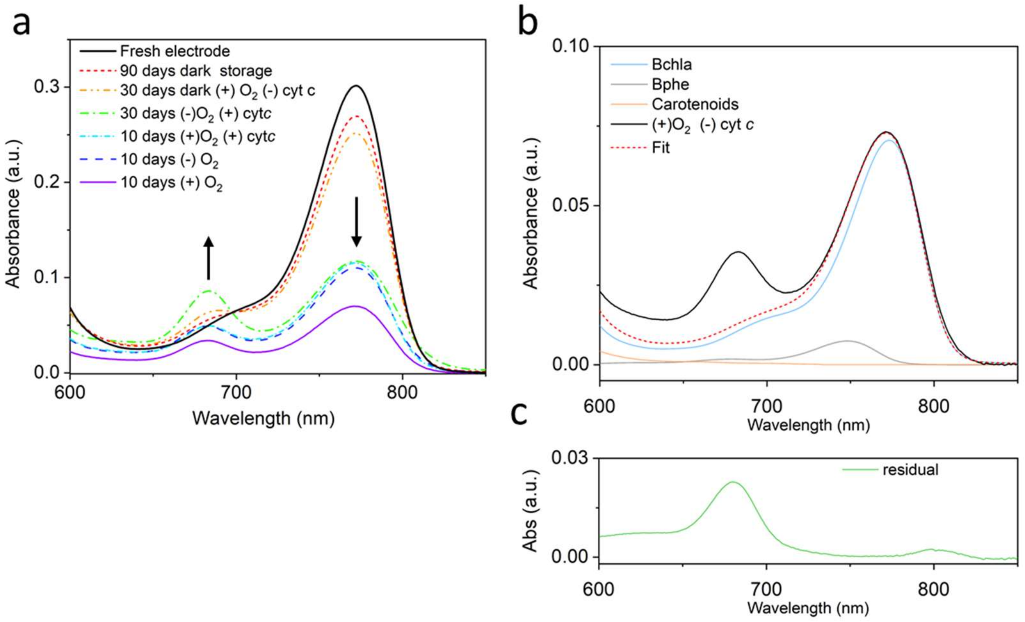

Figure S13. Bacteriochlorophyll degradation product characterization. (a) Absorbance spectra of pigments extracted in 80% acetone from a freshly prepared electrode (black solid), and electrodes incubated for 10-90 days under a variety of conditions. Arrows indicate the change in peaks over time. (b) Fit of the spectrum of extracted pigments with the spectra of BChl *a*, BPhe *a* and spheroidenone. (c) Residual from the fit in (b) showing the spectrum of the degradation product.

Supporting text to Figure S13. The loss of contributions from the LH1 BChls to EQE spectra (Figure S12a), and loss of LH1 contributions to 1-Reflectance spectra, was accompanied by an increased absorption around 680 nm (Figure S12b). Pigment extractions of electrodes exposed to prolonged illumination showed a clear drop in absorption from BChl *a* at 770 nm and a relative increase in a band centered at 680 nm (Figure S13a). A spectral deconvolution was carried out to isolate the spectrum of this 680 nm absorbing degradation product (Figure S13b). After a fit with the spectra of BChl *a* (major component), BPhe *a* (minor component – naturally present at one per 16 BChl *a*) and spheroidenone carotenoid (which absorbs weakly between 600 and 700 nm), a rather large residual component with a peak at 680 nm remained (Figure S13c), which is attributed to 3-acetyl-chlorophyll *a*[17–19]. This BChl *a* degradation product has an extinction coefficient estimated as being 10-fold smaller than that of BChl *a* in LH2[19], which explains why the loss of amplitude at 770 nm is not fully recovered at 680 nm. Another commonly reported BChl *a* degradation product via chemical oxidation with quinones is 2-desvinyl-2-acetylchlorophyll, with a similar absorption maximum around 680 nm[20,21]. As we did not see this in pigments extracted from an electrode incubated in the dark in the presence of quinone, we can exclude the chemical oxidation of BChl *a* by ubiquinone as a degradation pathway.

References

[1] V. M. Friebe, J. D. Delgado, D. J. K. Swainsbury, J. M. Gruber, A. Chanaewa, R. Van Grondelle, E. Von Hauff, D. Millo, M. R. Jones, R. N. Frese, *Adv. Funct. Mater.* **2016**, *26*, 285–292.

[2] D. Mersch, C. Y. Lee, J. Z. Zhang, K. Brinkert, J. C. Fontecilla-Camps, A. W. Rutherford, E. Reisner, *J. Am. Chem. Soc.* **2015**, *137*, 8541–8549.

[3] X. Fang, K. P. Sokol, N. Heidary, T. A. Kandiel, J. Z. Zhang, E. Reisner, *Nano Lett.* **2019**, *19*, 1844–1850.

[4] H. P. Permentier, K. A. Schmidt, M. Kobayashi, M. Akiyama, C. Hager-Braun, S. Neerken, M. Miller, J. Amesz, *Photosynth. Res.* **2000**, *64*, 27–39.

[5] V. M. Friebe, D. Millo, D. J. K. Swainsbury, M. R. Jones, R. N. Frese, *ACS Appl. Mater. Interfaces* **2017**, *9*, 23379–23388.

[6] M. Kamran, J. D. Delgado, V. Friebe, T. J. Aartsma, R. N. Frese, *Biomacromolecules* **2014**, *15*, 2833–2838.

[7] P. Qian, M. Z. Papiz, P. J. Jackson, A. a. Brindley, I. W. Ng, J. D. Olsen, M. J. Dickman, P. a. Bullough, C. N. Hunter, *Biochemistry* **2013**, *52*, 7575–7585.

[8] M. R. Jones, *Biochem. Soc. Trans.* **2009**, *37*, 400–407.

[9] M. Sener, J. Strumpfer, A. Singharoy, C. N. Hunter, K. Schulten, *Elife* **2016**, *5*, 09541.

[10] E. Y. Katz, A. Y. Shkuropatov, O. I. Vagabova, V. A. Shuvalov, *Biochim. Biophys. Acta - Bioenerg.* **1989**, *976*, 121–128.

[11] N. Lebedev, S. A. Trammell, A. Spano, E. Lukashev, I. Griva, J. Schnur, *J. Am. Chem. Soc.* **2006**, *128*, 12044–12045.

[12] M. J. Den Hollander, J. G. Magis, P. Fuchsenberger, T. J. Aartsma, M. R. Jones, R. N. Frese, *Langmuir* **2011**, *27*, 10282–10294.

[13] K. R. Stieger, S. C. Feifel, H. Lokstein, F. Lisdat, *Phys. Chem. Chem. Phys.* **2014**, *16*, 15667–15674.

[14] V. M. Friebe, D. J. K. Swainsbury, P. K. Fyfe, W. van der Heijden, M. R. Jones, R. N. Frese, *Biochim. Biophys. Acta - Bioenerg.* **2016**, *1857*, 1925–1934.

[15] S. a Trammell, A. Spano, R. Price, N. Lebedev, *Biosens. Bioelectron.* **2006**, *21*, 1023–8.

[16] E. Laviron, *J. Electroanal. Chem. Interfacial Electrochem.* **1979**, *101*, 19–28.

[17] W. S. Kim, *Biochim. Biophys. Acta - Biophys. Incl. Photosynth.* **1966**, *112*, 392–402.

[18] L. Limantara, P. Koehler, B. Wilhelm, R. J. Porra, H. Scheer, *Photochem. Photobiol.* **2006**, *82*, 770.

[19] Z. K. Makhneva, A. A. Ashikhmin, M. A. Bolshakov, A. A. Moskalenko, *Biochem.* **2016**, *81*, 176–186.

[20] T. N. Kropacheva, A. J. Hoff, *J. Phys. Chem. B* **2001**, *105*, 5536–5545.

[21] J. R. L. Smith, M. Calvin, *J. Am. Chem. Soc.* **1966**, *88*, 4500–4506.
